# Eco–Friendly Peelable Active Nanocomposite Films Designed for Biological and Chemical Warfare Agents Decontamination

**DOI:** 10.3390/polym13223999

**Published:** 2021-11-19

**Authors:** Gabriela Toader, Aurel Diacon, Traian Rotariu, Mioara Alexandru, Edina Rusen, Raluca Elena Ginghină, Florentina Alexe, Ramona Oncioiu, Florina Lucica Zorila, Alice Podaru, Andreea Elena Moldovan, Daniela Pulpea, Ana Mihaela Gavrilă, Tanta Verona Iordache, Raluca Șomoghi

**Affiliations:** 1 Military Technical Academy “Ferdinand I”, 39-49 George Cosbuc Boulevard, 050141 Bucharest, Romania; gabriela.toader@mta.ro (G.T.); podaru.alice04@gmail.com (A.P.); andreea.voicu89@gmail.com (A.E.M.); pulpea.daniela@gmail.com (D.P.); 2 Faculty of Applied Chemistry and Materials Science, University ‘Politehnica’ of Bucharest, 1-7 Gh. Polizu Street, 011061 Bucharest, Romania; aurel.diacon@upb.ro (A.D.); edina.rusen@upb.ro (E.R.); 3 Microbiology Laboratory of Horia Hulubei National Institute for R&D in Physics and Nuclear Engineering, 30 Reactorului St., 077125 Bucharest-Magurele, Romania; florina.zorila@nipne.ro; 4 Research and Innovation Center for CBRN Defense and Ecology, 225 Soseaua Oltenitei, 041327 Bucharest, Romania; raluca.ginghina@nbce.ro (R.E.G.); florentina.alexe@nbce.ro (F.A.); ramona.oncioiu@nbce.ro (R.O.); 5 National Institute of Research and Development for Chemistry and Petrochemistry, 202 Splaiul Independentei, 060041 Bucharest, Romania; ana.gavrila@icechim.ro (A.M.G.); tanta-verona.iordache@icechim.ro (T.V.I.); r.somoghi@gmail.com (R.Ș.); 6 Petroleum-Gas University of Ploiesti, 100680 Ploiesti, Romania

**Keywords:** eco-friendly, nanoparticles, chemical warfare agents, sulfur mustard (**HD**), dimethyl methylphosphonate (**DMMP**), biological agents, nanocomposite films, decontamination, antimicrobial materials, peeling, active coatings

## Abstract

In the context of imminent threats concerning biological and chemical warfare agents, the aim of this study was the development of a new method for biological and chemical decontamination, employing non-toxic, film-forming, water-based biodegradable solutions, using a nano sized reagent together with bentonite as trapping agents for the biological and chemical contaminants. Bentonite-supported nanoparticles of Cu, TiO_2_, and Ag were successfully synthesized and dispersed in a polyvinyl alcohol (**PVA**)/glycerol (**GLY**) aqueous solution. The decontamination effectiveness of the proposed solutions was evaluated by qualitative and quantitative analytical techniques on various micro-organisms, with sulfur mustard (**HD**) and dimethyl methylphosphonate (**DMMP**) as contaminants. The results indicate that the peelable active nanocomposite films can be successfully used on contaminated surfaces to neutralize and entrap the hazardous materials and their degradation products. Mechanical and thermal characterization of the polymeric films was also performed to validate the decontamination solution’s potential as peelable-film generating materials. The removal efficacy from the contaminated surfaces for the tested micro-organisms varied between 93% and 97%, while for the chemical agent **HD**, the highest decontamination factor obtained was 90.89%. **DMMP** was almost completely removed from the contaminated surfaces, and a decontamination factor of 99.97% was obtained.

## 1. Introduction

The most recent global crisis caused by the SARS-CoV-2 pandemic demonstrated that it is vital to be prepared for emerging sanitary, biological, chemical, or environmental hazards. Decontamination has always represented a major challenge, but especially now, in the current situation of the COVID-19 crisis, developing efficient methods for the neutralization and the removal of the contaminants should become a priority. Although chemical and biological weapons are forbidden by the Chemical Weapons Convention [1] and Biological and Toxin Weapons Convention [2], certain states are still suspected to currently possess chemical weapons, as well as biological weapons. Moreover, even if a virus, like SARS-CoV-2, is not on the list of biological weapons, it is still important to develop new methods for biohazards management. Considering the large number of nosocomial infections with pathogens resistant to classical methods of treatment and decontamination (with antibiotics and disinfectants), in recent years, the emphasis has been on the development of new, versatile products with antimicrobial properties. Another important threat is represented by the main ingredients used for the synthesis of chemical warfare agents (**CWA**): sulfur mustard, sarin, soman, tabun, Vx, etc., which are also found in the manufacturing processes of various chemical and pharmaceutical industries (chlorine, phosgene, and cyanides). The use of chemical and biological agents from military stockpiles or biological civilian applications drives the critical need to improve decontamination capabilities worldwide. Therefore, decontamination plays a crucial role in defense against biological and chemical warfare agents (BCWA). After a chemical or biological attack, decontamination is vital. This complex process converts hazardous materials into products that can be safely handled. The methods that are typically applied are nucleophilic reactions or oxidations [3]. Toxic chemicals or micro-organisms must be eliminated by the application of efficient decontamination methods as quickly as possible in order to be able to resume routine activities. For military purposes, decontamination is undertaken to restore the combat effectiveness of equipment and personnel as rapidly as possible [4]. Most current decontamination procedures are labor- and resource-intensive, require excessive amounts of water, are corrosive and/or toxic, and are not considered environmentally safe [4,5,6,7]. Current research and development are focused on developing decontamination systems that would overcome these limitations and effectively decontaminate a broad spectrum of chemical and biological agents (CB agents) from all surfaces and materials [6,8,9,10,11]. There is no single technology that will be applicable in all situations and all types of contaminations because the nature and extent of contamination are different in different places. Surface decontamination is very difficult to achieve as contaminants can be located within the pores and cracks of materials, which makes their removal more challenging [12]. Depending on the type of contaminating agent, many decontamination methods for surfaces can be found in the literature: aspiration [13], abrasion of the surface layer [14], rinsing with water or with solvents [15], foams [15,16], gels [16], polymeric coatings [17,18,19], etc. The main disadvantages and limitations of the existing surface-decontamination solutions are: most of them are corrosive and/or toxic, affecting the decontaminated substrate and also exposing the user to hazardous materials; most of the existing decontamination methods are not considered environmentally safe because they require excessive amounts of reactants, solvents, or water, generating enormous quantities of post-decontamination waste, which requires subsequent decontamination; the decontamination systems that require large amounts of water do not represent a feasible solution because water can often be difficult to find (for example, on the battlefield [4]), and the disposal of this contaminated water will also further represent a cumbersome problem. Recent trends in BCWA decontamination technologies involve the use of materials capable of neutralizing contaminants under atmospheric conditions via hydrolysis and/or oxidation routes, under mild conditions of the reaction. Besides the classical decontamination methods, the use of modern methods that imply using polymeric films/coatings seems to bring multiple advantages for BCWA removal [11,17,20]. In comparison with the traditional techniques, these decontamination methods usually consist of applying a smaller amount of material (containing the active ingredients) onto the contaminated surface, thus resulting in a coating that will entrap and neutralize the contaminant and can be easily removed and compactly stored at the end of the decontamination process. This new decontamination method can be found referred to in that literature as “stripping/peelable coating techniques” [5,11,17,18,20]. This technique applies to a wide range of contaminants and surfaces/materials. Thus, polymeric peelable films represent a modern and versatile method for surface decontamination [6,10,14,21,22]. Various polymers can be listed as film-forming polymers: acrylates [17,21,23,24], silicones [25], vinyl polymers [12,26,27], polyurea [28,29,30,31,32], alginate derivates [17,33,34,35]. Film-forming materials are already commercially available as products comprising paint-like polymeric mixtures that can be applied by spray-on or roll-on/brush-on techniques, and they can form peelable coatings for decontamination/decommissioning purposes: CBI Polymers, New York, USA—DeconGel™ 1108, Instacote Inc Protective Coatings, Erie, USA—InstaCote™ CC Wet/CC Strip, or Bartlett Nuclear Inc., Plymouth, USA—StripCoat TLC Free™. Although these products do generate a smaller volume of secondary waste and they ensure reasonable DFs, their main disadvantage is represented by the toxicity and corrosivity of some of the active ingredients comprised in these commercial formulations. 

The development of nanotechnology in various fields has experienced exponential growth over the last decade. In the biological and chemical decontamination fields, formulations based on nanoparticles and metal oxide nanoparticles have attracted a tremendous interest due to their remarkable properties. Small particle sizes and high specific surface areas bring multiple advantages and unique physicochemical properties that facilitate the adsorption and degradation of toxic compounds [36,37]. Advances in the preparation of metallic NPs and metallic oxides like ZnO, MgO, CaO, CeO_2_, ZrO_2_, TiO_2_, etc., have led to the development of a new class of antimicrobial materials and decontaminants for chemical warfare agents with a high stability under harsh process conditions [8,37]. Highly ionic metallic NPs (e.g., Cu-NPs, Ag-NPs) are of particular interest due to their numerous reactive surface sites with atypical crystal morphologies. Ag and Cu nanoparticles immobilized on metal-oxide substrate have been demonstrated to neutralize viruses, bacteria, and fungi [38]. Nano-scaled copper particles (Cu-NPs) have many applications in industry, such as in gas sensors, high-temperature superconductors, solar cells, and other applicatoin. Copper ions have demonstrated antimicrobial activity against a wide range of micro-organisms (*Staphylococcus aureus*, *Salmonella enteric*, *Campylobacter jejuni*, *Escherichia coli*, and *Listeria monocytogenes*) [39]. The antibacterial effect exhibited against bacterial cell functions can occur through various mechanisms, depending on the physicochemical properties of NPs and the type of interactions between bacterial cells (e.g., adhesion to s Gram-negative bacterial cell wall due to electrostatic interaction [39]). These interactions lead to a disruption of the integrity of the bacterial membrane and finally cause the death of the micro-organism. Copper NPs possess better properties in comparison with other expensive metals with antimicrobial activity, such as silver and gold [40]. Silver nanoparticles (Ag-NPs) are known to neutralize both bacteria and viruses through metal-ion binding. In 2003, during the first SARS outbreak, Al_2_O_3_-supported Ag was investigated for the neutralization of SARS coronavirus, *E. coli* (bacterium), and *Debaryomyces polymorfus* (fungi). After only five minutes of exposure to the Ag nanoparticles, the three pathogens were inactivated successfully. The mechanism was not investigated, but it is assumed that catalytic oxidation is responsible and not metal poisoning (Au and Cu inactivate bacteria, viruses, and fungi only under aerobic conditions) [38].

Metal NPs and metal oxide NPs are also efficient for the decontamination of chemical warfare agents [41,42]. Sulfur mustard (**HD**) can be decontaminated through dichlorination, oxidation, or hydrolysis mechanisms, thus being converted into non-toxic products [41]. From all the materials used for the chemical degradation of **HD**, it was demonstrated that nano-oxides can adsorb and degrade sulfur mustard to thiodiglycol and divinyl sulfide at room temperature. A disadvantage of this decontamination method is that requires several hours for full degradation [42]. There are also studies regarding metal-organic frameworks (MOFs) constructed from metal ions or clusters and multifunctional organic linkers through self-assembly, which have been reported as perfect candidates for chemical and biological decontamination. The most well-known is Cu-BTC MOF, and it was also demonstrated to be capable of hydrolyzing **HD** and nerve agents under ambient conditions via its coordination of water molecules, which have an important practical value [43,44]. Silver nanoparticles (Ag-NPs) encapsulated in MOF were reported as efficient decontaminants for **HD** [43].

While the abovementioned BCWA decontamination methods offer promising possibilities, they also possess a series of disadvantages, such as high production costs, laborious production processes, toxicity and corrosivity of some of the active ingredients, generation of a large amount of post-decontamination waste, unsatisfying decontamination degrees, etc.

The novelty of this paper consists in the development of a new method of biological and chemical decontamination by employing non-toxic, film-forming, water-based biodegradable solutions using both neutralization and adsorption mechanisms for the removal of the contaminant from a surface by employing a nanosized reagent together with bentonite as trapping agents for BCWA contaminants. Once they are applied to the contaminated surface, the neutralization of the contaminants occurs, followed by their entrapment in the polymer-clay system. After drying, these solutions form strippable films that can be easily removed from the surface. Decontamination tests herein reported confirmed the antimicrobial activity of the decontamination solutions (DF ≥ 93%) and the successful neutralization and removal of chemical agents: up to 90% decontamination efficiency for **HD** and over 99% decontamination efficiency for dimethyl methylphosphonate. Therefore, this study reveals that BC contaminants were successfully neutralized and entrapped in the polymer matrix, demonstrating that this novel ecological approach towards obtaining innovative peelable active nanocomposite films for the removal of biological and chemical agents from contaminated surfaces could represent a powerful environmentally responsible tool for decontamination applications in the future.

## 2. Materials and Methods

### 2.1. Materials

Poly(vinyl alcohol) (**PVA**, 86.7–88.7% hydrolysis degree, Mw ≈ 130,000 Da, DP ≈ 2700, Sigma–Aldrich), hydrophilic bentonite (**BT**, Nanomer^®^ PGV, Sigma–Aldrich, St. Louis, MO, USA), anhydrous glycerol (**GLY**, Sigma–Aldrich, St. Louis, MO, USA), copper(II) acetate monohydrate (≥98%, Sigma Aldrich, St. Louis, MO, USA), titanium(IV) oxide (nanopowder, <100 nm particle size, 99.5% trace metals basis, Sigma Aldrich, St. Louis, MO, USA), Triton™ X-100 solution (Sigma Aldrich, St. Louis, MO, USA), ascorbic acid (≥99%, Sigma Aldrich, St. Louis, MO, USA), Silver nitrate (≥99.0%, Sigma Aldrich, St. Louis, MO, USA), tri-sodium citrate dihydrate (Sigma Aldrich, St. Louis, MO, USA), and sodium borohydride (≥99.0%, Sigma Aldrich, St. Louis, MO, USA) were used as received. For the chemical decontamination tests, real chemical warfare agents (**CWA**) were used: bis(2-chloroethyl) sulfide (**HD,** sulfur mustard, purity: 95%, own synthesis), together with a chemical warfare simulant: dimethyl methylphosphonate (**DMMP**, as simulant for nerve agents, ≥97%, Sigma Aldrich). All the tests involving the decontamination of the toxic agents utilized in this study were performed at the Research and Innovation Center for CBRN Defense and Ecology in the ‘Chemical Analysis Laboratory’ from Bucharest, the only OPCW-designated laboratory in Romania.

### 2.2. Methods

#### 2.2.1. Preparation of Decontamination Solutions

Solutions free of metal nanoparticles and bentonite were initially prepared to serve as reference points (Table 1). The BCWA decontamination solutions based on bentonite-supported metal nanoparticles were obtained as follows: the metallic salts were dissolved in water (according to Table 2), various amounts of bentonite and TiO_2_ were dispersed in these solutions (continuous magnetic stirring, 800 rpm), and the nanoparticle precursor and bentonite (or bentonite and TiO_2_) were kept in contact under stirring for 24 h. After this, the obtained dispersions were sonicated for 30 min while the corresponding reducing agents (according to Table 2 and Table 3) were added. **PVA** was introduced next, and the dispersions were maintained at 95 °C under vigorous stirring until the complete dissolution of the polymer. Finally, the glycerol was added last, while the dispersions were allowed to cool down.

#### 2.2.2. Preparation of the Nanocomposite Films

The obtained solutions were further used for decontamination tests, but they were also employed for obtaining square-shaped, thin nanocomposite films that were useful for characterization through different analytic procedures. To obtain the nanocomposite films by the casting method, approximately 100 mL of each decontamination solution was introduced in a square (12 cm × 12 cm) glass mold, placed on a perfectly flat surface, and allowed to dry (at 25 °C, 50–55% relative humidity). Afterwards, the films obtained were peeled and employed for further investigation.

### 2.3. Decontamination Tests

#### 2.3.1. Biological Decontamination Tests

The biological decontamination tests involved the characterization of the antimicrobial activity of the decontamination solutions, followed by the evaluation of the efficacy of the strippable nanocomposites for the removal of biological contaminants from the targeted surfaces. For the characterization of antimicrobial activity of these substances, three of the most used methods appropriate for this type of sample were applied: minimal inhibitory concentration (MIC), minimal bactericidal concentration (MBC) and time-kill test. The MIC value is defined as the lowest concentration of the antimicrobial agent that inhibits the visible growth of the micro-organisms tested. This is usually expressed in mg/mL or mg/L. The MBC is defined as the lowest concentration of antibacterial agent needed to kill 99.9% of the final inoculum after incubation for 24 h under a standardized set of conditions. Time-kill assay is the most appropriate method for determining bactericidal effect. It is a good method for obtaining information about the interaction between the antimicrobial agent and the microbial strain. The time-kill test reveals a time-dependent or a concentration-dependent antimicrobial effect [45].

##### Minimal Inhibitory Concentration

The antimicrobial activity of the decontamination solutions was evaluated against *Staphylococcus aureus* (ATCC 6538) as a model for Gram-positive bacteria and *Escherichia coli* (ATCC 8739) and *Pseudomonas aeruginosa* (ATCC 9027) as a model for Gram-negative bacteria. *S. aureus*, *E. coli*, and *Ps. aeruginosa* were chosen, considered standard micro-organisms for testing the antimicrobial properties of newly synthesized products [46]. After cultivation overnight in Muller Hinton broth (MHb) (Merck) at 37 °C with stirring (200 rpm), the bacterial strains were harvested. Portions of suspension were harvested by centrifugation and resuspended in phosphate buffer saline (PBS) (Sigma-Aldrich, St. Louis, MO, USA). The suspensions were adjusted to approximately 10^6^ CFU/mL [47]. Minimum inhibitory concentrations (MIC) were established for each solution by the broth microdilution method [48,49]. Two-fold serial dilutions of each solution were performed in MHb in duplicate. Negative and positive controls were associated [50]. The inhibitory effect of the substances was evaluated starting from 50% concentration (the samples of substances were diluted 1:1 with MHb). A total of 10 µL of the micro-organism suspensions (~10^4^ CFU) was added in each well corresponding to the testing samples and controls. Because the solutions are turbid themselves and the bacterial growth is difficult to discern, at the end of the incubation period, 10 µL of resazurin 0.1% was added to each well. After 2 h of incubation with resazurin, the plates were read.

##### Minimal Bactericidal Concentration

MBC was determined after broth microdilution by subculturing the content of each well that did not show any visible signs of growth on the surface of non-selective agar plates (Muller-Hinton agar, MHa, Merck, Darmstadt, Germany). This allowed for the determination of the number of surviving cells (CFU/mL) after 24 h of incubation at 37 °C [45].

##### Time-Kill Test

Portions of more concentrated bacterial strain suspensions (10^7^ CFU/mL), prepared as previously described, were treated with the studied substances at 2× MIC concentration for solutions with MIC values established and 50% concentration for the others and kept in direct contact for 2 h and 24 h, respectively, at 37 °C. At each established time, portions of bacterial cultures were serial diluted in PBS and then plated on Muller Hinton agar medium (MHa). After incubation at 37 °C for 24 h, bacterial survival was evaluated [51].

##### Efficacy of Biological Contaminant Removal from the Targeted Surfaces

The efficacy of micro-organism removal from the surface was determined by calculating the decontamination factor achieved by polymeric nanocomposite films after exfoliation. Sterile surfaces (sterile Petri dishes) were contaminated with known concentrations of micro-organisms. Two micro-organisms were applied separately: *Staphylococcus aureus* (ATCC 6538) and *Escherichia coli* (ATCC 8739). The contaminated plates were dried, and then the polymer solutions were applied. The plates were kept under airflow at room temperature. After 24 h, the formed polymeric films were exfoliated. After this, the culture media was applied by spreading on the decontaminated surfaces of Petri dishes. The plates were incubated at 37 °C for 24 h. After the incubation period, the number of CFUs was counted. DF was calculated by considering the initial contamination and the number of CFUs counted after the incubation period.

#### 2.3.2. Chemical Warfare Agent Decontamination Tests

Chemical decontamination using one real warfare agent, sulfur mustard (Yperite or **HD**), and one simulant for nerve agents, dimethyl methylphosphonate (**DMMP**), was performed as follows: firstly, controlled contamination was performed, followed by the application of the decontamination solution on the contaminated surface (after 5 min from contamination); following the film-curing process (20–24 h), the nanocomposite films containing the degradation products of **HD** and **DMMP** were peeled off and subjected to extraction in DCM, followed by GC-MS analysis.

In the first step (controlled contamination, 10 g_(toxic)_/m^2^), some metallic probes, measuring 10 cm^2^, were contaminated with 7.87 µL (10 mg) of **HD** (ρ = 1.27 g/cm^3^) and other metallic probes, also measuring 10 cm^2^, were contaminated with 8.61 µL (10 mg) of **DMMP** (ρ = 1.145 g/cm^3^). After 5 min of toxic-metallic probe direct contact, approximately 1.5 mL of decontamination solution was poured over the contaminated area, making sure the surface was completely covered by the liquid. Once the decontamination solution was placed on the contaminated surface, the active ingredients set up the degradation of the toxic agent while being adsorbed by bentonite nanoclay and entrapped in the polymeric matrix of the nanocomposite. After approximately 20 h, the polymeric nanocomposite coating containing the entrapped contaminants could be easily peeled off. Both the decontaminated surface and the film obtained were subjected to extraction in 10 mL of dichloromethane (DCM). The organic extracts were dried over sodium sulfate, filtered on 45 µm a Sartorius filter, and analyzed by the GC-MS technique. To evaluate the decontamination efficiency, the decontamination factor (DF) was calculated by considering the initial concentration of contaminant and the residual toxic found on the metallic probe after decontamination.

DF = 100 × (C_0_ − C_f_)/C_0_ (1)

where DF is the decontamination factor, C_0_ is the initial toxic concentration, and C_f_ is the final concentration, reflecting the residual contamination. Measurements were repeated in triplicate, and the average values obtained were reported.

### 2.4. Characterization

To acquire quality imaging of the samples, a high-resolution transmission electron microscope (HRTEM), type TECNAI F30 G2STWIN, Fei Company, Oregon, USA, was used at 300 kV acceleration voltage and with a resolution of 1 Å. The correlation between dynamic viscosity and shear gradient of the decontamination solutions was studied to establish a model flow profile of the polymeric solution with superior film-forming characteristics. Rheological tests were performed on a Rheotest 2.1 device (Rheotest Medingen GmbH, Ottendorf-Okrilla, Germany) with coaxial cylinders at room temperature (25 °C) to determine the behavior of the solutions. The amount of solvent evaporated in time at different temperatures (25 °C, 30 °C, and 35 °C) was used to investigate the drying profile of the nanocomposite films. An ATS 120 Axis Thermobalance was used to measure the evaporation rate of 4 mL of sample for evaluation of the film-formation process. Promas software calculated the evaporation rate by weighting the sample every 150 s. FT-IR spectra were obtained using a Perkin Elmer Spectrum Two (Perkin Elmer, Waltham, MA, USA) with a Pike MiracleTM ATR modulus and a 4 cm^−1^ resolution, from 550 to 4000 cm^−1^. To investigate the mechanical proprieties, polymeric films were obtained by casting method and then cut in a dumbbell shape with 75 mm overall length and a narrow section of about 25 ± 1 mm, which were subsequently subjected to tensile tests on a 710 Titan 2 universal strength-testing machine equipped with a 3000 N force cell, according to ISO 37: 2011(E). The test involves continuous observation of the length and force variation with an accuracy of ±0.2% at a speed of 8.33 mm/s. To compare the results, the mean values of each sample were plotted in a stress/strain graph. Five specimens from each sample were subjected to tensile tests. Samples weighing approximately 25–30 mg were subjected to thermal tests, heated from 30 °C to 450 °C with a constant heating rate of 5 °C/min on a DTA OZM 551 Ex Differential Thermal Analysis System equipped with Meavy dedicated software. GC-MS investigations were performed on a GC Thermo Scientific Trace 1310 (Thermo Fisher Scientific, Waltham, MA, USA) gas chromatograph coupled with a TSQ 9000 triple quadrupole mass spectrometer (MS/MS) (Thermo Fisher Scientific, Waltham, MA, USA) using a TR5MS GOLD capillary column (5% phenyl 95% dimethylpolysiloxane). The injection mode used was splitless with an injector temperature of 250 °C and helium as carrier gas (1.5 mL/min). The temperature program started from 40 °C, up to 300 °C, with a rate of 10 °C/minute. Electron impact ionization (EI) mode (mass range between 40 and 650 amu) was used. The compounds were identified based on the interpretation of MS/EI fragmentation.

## 3. Results and Discussion

This first step of this study consisted of the synthesis of bentonite-supported metal and metal oxide nanoparticles that are suitable for decontamination applications, capable of reacting with chemical warfare agents to form non-toxic products while also neutralizing biologic agents due to their anti-bacterial properties. Thus, nanosized hydrophilic bentonite was used as support for the metal/metal oxides nanoparticles during their generation. In the decontamination process, bentonite can act as an efficient adsorbent for the contaminants, facilitating their deactivation induced by the presence of the nanoparticles. The bentonite-supported metal/metal oxide nanoparticles were dispersed in an aqueous solution of polyvinyl alcohol (**PVA**), a biodegradable polymeric matrix, which plays an essential role in holding together (and thus “binding”) all the components of the decontamination solution (including the entrapped contaminant). The excellent film-forming capacity of **PVA** ensures the formation of peelable films from these decontamination solutions, which facilitates the efficient removal of contaminants from different types of surfaces.

**Figure 1:**
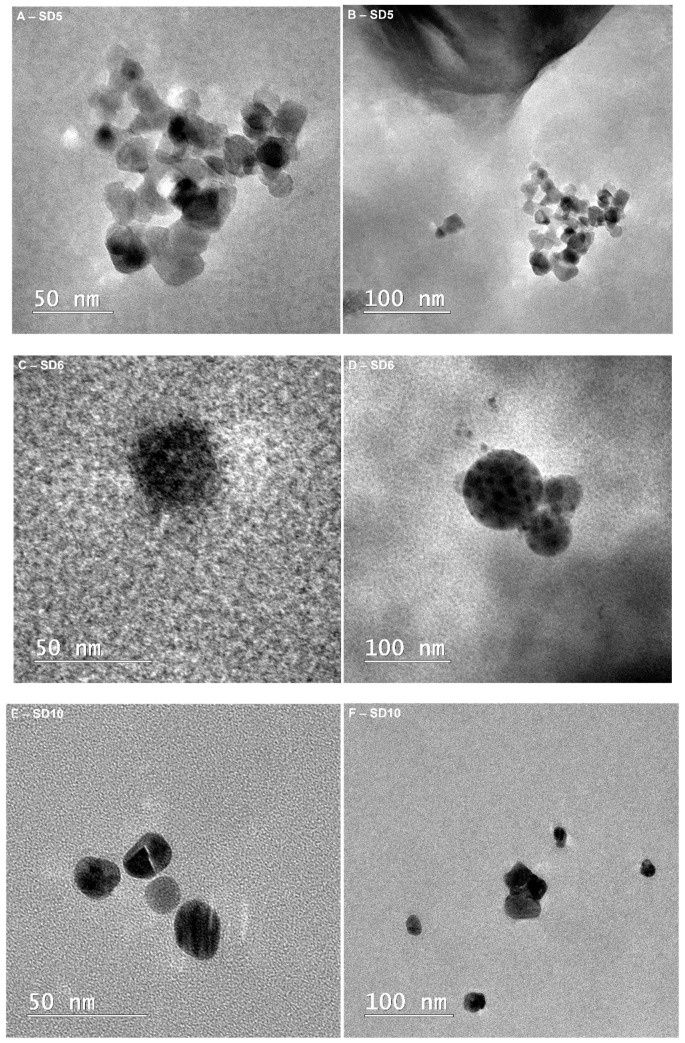

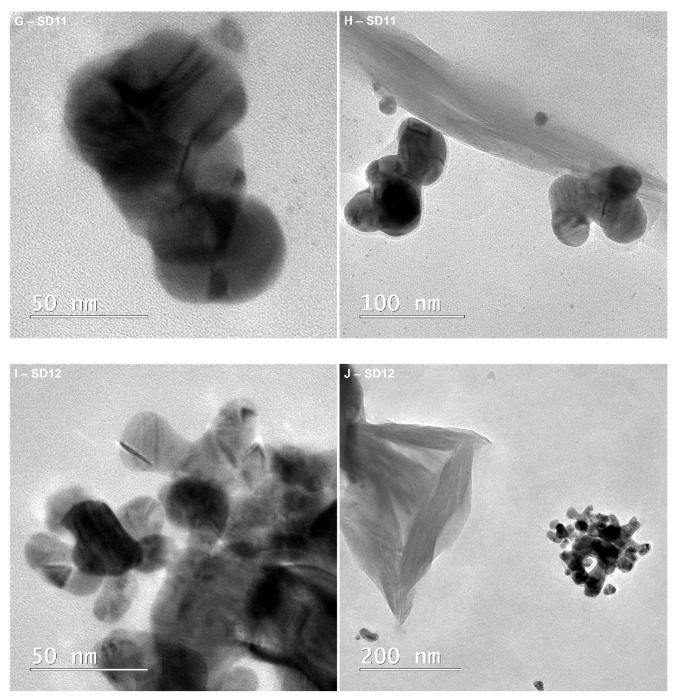
TEM image of bentonite-supported metal nanoparticles employed in the decontamination solutions: (**A**,**B**) SD5-mag 100,000×, mag 50,000×; (**C**,**D**) SD6-mag 180,000×, mag 50,000×; (**E**,**F**) SD10-mag 180,000×, mag 50,000×; (**G**,**H**) SD11-mag 180,000×, mag 60,000×; (**I**,**J**) SD12-mag 180,000×, mag 29,000×.

TEM analysis was employed as the first characterization tool to confirm the generation of Cu and Ag nanoparticles in the decontamination solutions (Figure 1). From the images presented in Figure 1A–D, it can be noticed that for the samples containing 1% bentonite (SD5), the CuNPs diameter is around 20 nm, while for the SD6 (containing 1.5% bentonite), the particle size is around 40–60 nm. In the case of SD4 (0.5% bentonite), the presence of independent nanoparticles outside the clay structure was not visible by TEM. Thus, as the concentration of bentonite increased in the samples, larger nanoparticles were observed. Similar behavior was evidenced for the AgNPs (samples SD10, SD11, and SD12). The particle size of the AgNPs increased at a higher clay content. An explanation for the particle size modification can be attributed to the adjustment of the growth process due to the presence of clay, which causes smaller particles to destabilize, due to the clay’s inherent electrostatic charge [52], and promotes growth. In the case of the samples containing TiO_2_, no visible effect on the particle size was observed; the CuNPs’ particle size varied, depending on the bentonite content.

**Figure 2:**
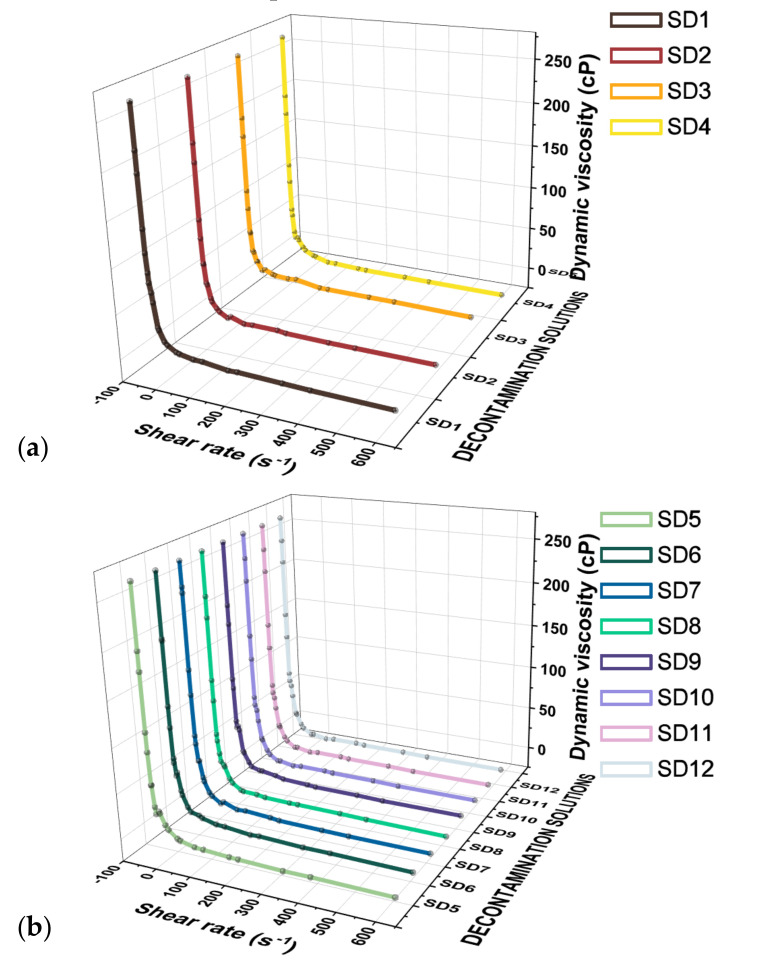
Variation of the dynamic viscosity with shear rate for the decontamination solutions, (**a**) SD1, SD2, SD3, SD4 and (**b**) SD5, SD6, SD7, SD8, SD9, SD10, SD11, SD12.

An important parameter that influences both the polymer solution deposition procedure and the type of surface that will undergo decontamination is represented by the dynamic viscosity of the solution. Thus, the solutions presenting a higher viscosity are suitable for application by brush technique, whereas less viscous solutions can be deposited by spray technique. A higher viscosity can also affect the mobility of the molecules influencing the rate of adsorption of the biological compounds, as well as decrease the capacity of the solution to enter the pores and cracks of the surface. Thus, the influence of each component on the viscosity of the solutions was evaluated, and the results are illustrated in Figure 2. Polyvinyl alcohol (**PVA**) has an essential role in film formation (SD1), while the addition of glycerol (SD2) improves the elasticity of the peelable films and bentonite aids in the improvement of the biological/chemical-agent retention inside the film by complexation and adsorption processes (SD3). The effect of glycerol and bentonite on the viscosity of the solution can be explained by the physical interactions that occur between the polymer and these components, such as the formation of hydrogen bonds. Considering these aspects, all the components employed can form hydrogen bonds with water or **PVA**, thus connecting the macromolecules through physical interaction. Consequently, there is an apparent increase in the molecular weight of the polymer, which leads to an increase in the viscosity of the decontamination solution. Additionally, the variation of the dynamic viscosity of the decontamination solutions containing copper and silver nanoparticles was investigated. Compared with the control sample (SD3), it can be noticed that for all the solutions, similar dynamic viscosity values were obtained, due to the low concentration of nanoparticles.

In order to determine the time required for solvent evaporation from the decontamination solution and film formation, determination of the evaporation rate is essential. The capacity of solvent evaporation (water in this case), after solution deposition, depends on several factors: temperature, humidity, type of surface, etc. but also on the viscosity and nature of solution constituents. To ascertain these values, parameters such as sample surface and quantity of solution were kept constant. The evaporation parameters are presented in Table S1. As the temperature is increased, the solvent evaporation rate is more predominantly influenced by the chemical composition of the solution, the interaction between the components, and solution viscosity. The measurements taken at temperatures between 25 and 40 °C showed that the solution’s components influence the evaporation rate, which is due to the intermolecular interaction between the components and water molecules; thus, the stronger the interaction, the longer the interval required for drying.

**Figure 3:**
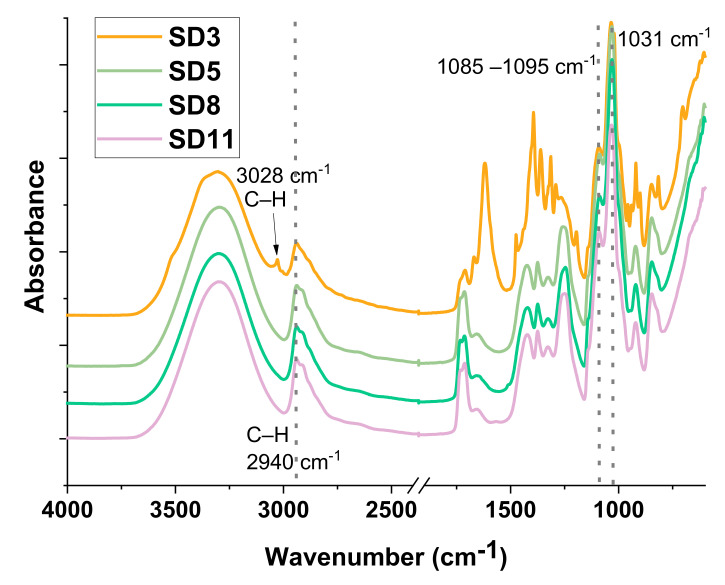
FT-IR spectra of the polymer films (SD3, SD5, SD8, and SD11).

FT-IR spectroscopy was employed to highlight the formed hydrogen bond between the polymer and glycerol, respectively, as well as the presence of bentonite in the polymer films. The results are presented in Figure 3 and detailed in Figure S1. The broad peaks around 3300 cm^−1^ can be attributed to hydroxyl groups from the **PVA** chain, while the values in the range of 1095–1085 cm^−1^ indicate the presence of hydrogen bonds formed between **PVA** and glycerol. The weak bands at 3028 and 2919 cm^−1^ can be assigned to C–H stretching vibrations. The strong peak highlighted around 1031 cm^−1^ has a double meaning: it appears due to the strong vibrations of the C–O bonds of a primary alcohol, and at the same time, it also indicates the presence of Si–O bonds due to bentonite clay. At the same time, an increase in absorbance is observed with the addition of bentonite.

**Figure 4:**
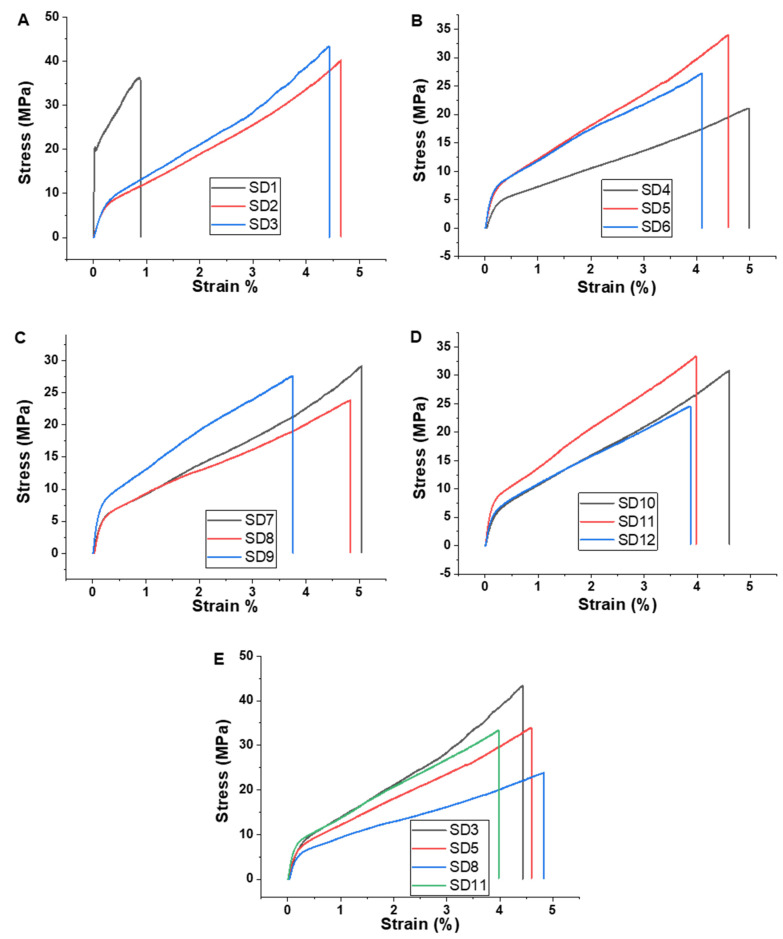
Tensile tests results for the polymer films: (**A**) SD1, SD2, SD3; (**B**) SD4, SD5, SD6; (**C**) SD7, SD8, SD9; (**D**) SD10, SD11, SD12; (**E**) SD3, SD5, SD8, SD11.

The mechanical properties of polymer films are very important for the peeling process and efficient decontamination of surfaces, which requires a high resistance but also a certain degree of elasticity. Figure 4 illustrates comparative stress-strain plots for all the nanocomposites obtained. As can be observed, the addition of glycerol led to higher stress-strain values. In the absence of glycerol, films containing only polyvinyl alcohol (SD1) are more resistant, but they are much too rigid and brittle to be used for surface decontamination. Based on the results of the tensile strength tests, it can be stated that each component modifies the mechanical properties of the films. Moreover, this aspect must be considered when formulating decontamination solutions to obtain the desired characteristics. Thus, to obtain films easily exfoliated from the surface of interest while avoiding fracture of the composite material, careful selection of components and their ratio is required. The exact values of the mechanical parameters are given in the Table S2. As can be observed, the nanocomposites containing metallic nanoparticles also displayed good mechanical properties. The polymeric films maintained their integrity after the completion of peeling. Thus, when these materials were subjected to low stretching forces (typical for a peeling process) the nanocomposite film had enough mechanical resistance and did not break. The mechanical resistance of the nanocomposites employed for surface decontamination is afforded by the synergistic effect between the reinforcing nanoclay, the polymer matrix, and the glycerol (acting as plasticizer).

**Figure 5:**
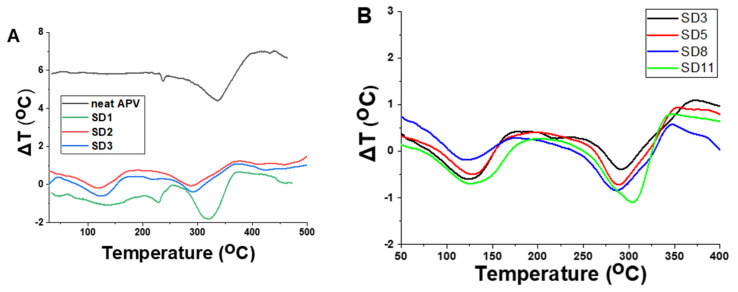
DTA thermograms for the polymer films (**A**) neat APV, SD1, SD2, SD3 and (**B**) SD3, SD5, SD8, SD11.

The differential thermal analysis presented in Figure 5 allowed the evaluation of the thermal characteristics of the polymer nanocomposite films. The thermal behavior of the film containing only **PVA** (**SD1**) is slightly different than that of the films containing glycerol. Thus, the peak situated at approximately 236 °C can be attributed to the melting of the crystalline regions/domains of **PVA**. Comparing SD2 and SD3, very small differences in terms of thermal transitions can be noticed. The first characteristic signal is endothermic for a temperature range between 70 and 150 °C (Figure 5A), which can be attributed to the evaporation of water trapped between the polymer chains. The shift of this peak to slightly higher values in the case of SD3 can be explained by the increased interaction due to the presence of bentonite. The second signal between 175 and 260 °C, also endothermic, can be attributed to the melting of the polymer (T_m_), while the signals after 270 °C can be attributed to the polymer degradation process. Similarly, the presence of bentonite (SD3), copper nanoparticles (SD5, SD8), or silver nanoparticles (SD11) in the composition of the polymer films leads to a slight modification of the characteristic temperature response; nevertheless, the responses of all samples are within the abovementioned temperature intervals. The decrease in melting temperature could be attributed to an increase in the thermal conductivity and polymer chain mobility due to the presence of the metallic nanoparticles (Table S3).

### 3.1. Decontamination Tests

**Figure 6:**
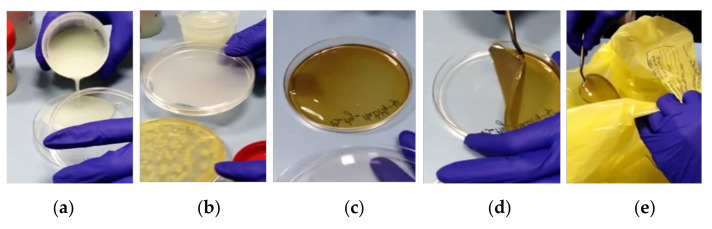
Decontamination using eco-friendly active nanocomposite peelable coatings: (**a**) decontamination solution, (**b**) decontamination solution is allowed to neutralize the contaminant, (**c**) dried peelable film, (**d**) peeling process and (**e**) decontamination waste.

The decontamination tests were performed to prove and evaluating the efficacy of this BCWA decontamination method. Biological decontamination tests were performed first, using *E. coli*, *Ps. aeruginosa* and *S. aureus* (as simulants for biological agents), and they were followed by chemical decontamination tests run on one real chemical warfare blistering agent, **HD**, and one simulant for neuroparalytic agents, **DMMP**. Figure 6 illustrates the steps taken to perform the decontamination using the herein-reported film-forming solutions.

The method for biological or chemical decontamination consists of the utilization of the synthesized eco-friendly active solutions (containing bentonite-supported nanoparticles) for the degradation/neutralization and entrapment of toxic agents, followed by the exfoliation of the formed film, which contains the degradation products resulting from the neutralization of the targeted hazardous materials.

### 3.2. Biological Decontamination Tests

**Figure 7:**
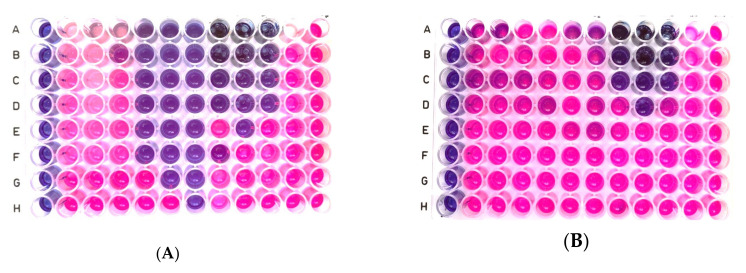
((**A**) *S. aureus* ATCC 6538) MIC determination against *S. aureus* ATCC 6538 observed from broth microdilution assay using MH broth and resazurin (columns from left to right: NC, SD4, SD5, SD6, SD7, SD8, SD9, SD11, SD10, SD12, SD3 (Bk), PC). and (**B**) *E. coli* ATCC 8739.observed from broth microdilution assay using MH broth and resazurin (columns from left to right: NC, SD4, SD5, SD6, SD7, SD8, SD9, SD11, SD10, SD12, SD3 (Bk), PC).

The results obtained of decontamination tests performed on biological contaminants are further detailed. An antimicrobial activity assay generated the MIC and MBC values of the decontamination solutions, displayed in Table 4 and Figure 7A,B. Solutions SD4, SD5, and SD6 revealed the lowest antimicrobial activity against bacterial strains used in the test. Solutions SD7, SD8, and SD9 showed low antimicrobial activity against Gram-negative bacteria (*E. coli*, *Ps. aeruginosa*) and pronounced activity against Gram-positive bacteria (*S. aureus*), MBC being established in this case. Solutions SD10, SD11, and SD12 showed stronger antimicrobial activity against Gram-negative bacteria, with MBC values established, but lower than those presented for Gram-negative bacteria for solutions SD7, SD8, and SD9.

The inhibition of the bacterial strain growth could be explained by specific interactions of nanoparticles with the cell envelope of micro-organisms [53]. When nanoparticles are small enough, they can penetrate membrane pores. Nanoparticles that can enter the cell membrane interact with bacterial enzymes, damaging the cell [54]. Some nanoparticles interact electrostatically with the bacterial membrane, and reactive oxygen species are generated, leading to the disruption of the membrane and DNA damages [55].

**Figure 8:**
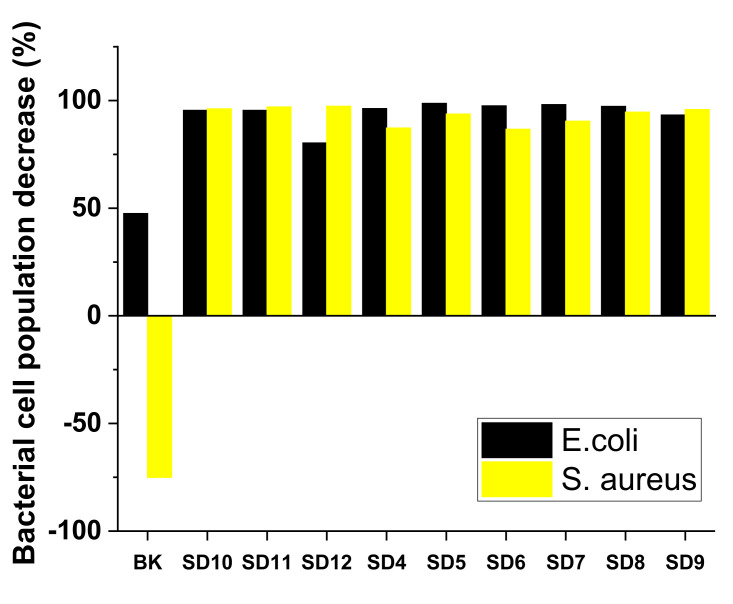
Bacterial cell population decrease (%) after 2 h of contact with decontamination solutions.

A time-kill assay was performed on *E. coli* and *S. aureus*. After the proposed contact times (2 h and 24 h) between strains and bentonite-supported nanoparticle solutions, bacterial growth was evaluated. In the case of the time-kill assay, it is observed that after 2 h of contact, all the decontamination solutions showed activity against both micro-organisms. After periods longer than 2 h of contact, in the case of *E. coli* strains, an increase in the number of CFU/mL was observed. Most likely, in the case of this bacteria, nanoparticles adhered to the surface of the cells or penetrated inside the membranes and were blocked. It is known that nanoparticles containing Cu, Zn, and Ti ions bind to negatively charged membranes (such as *E. coli*) [56]. This would explain the survival rate and resumption of the growth and division cycle. Considering that after 2 h of contact, an accelerated increase in the number of CFU/mL was observed, only the values recorded at time t1 (2 h of direct contact) were represented graphically (the results are illustrated in Table 5, Figure 8, and Figure S3), highlighting their antimicrobial activity during the short contact period. In the case of S. aureus, the activity is more pronounced, with observed antimicrobial activity even after 24 h in the case of SD7, SD8, SD9, SD10, SD11, and SD12 (results illustrated in Figure S4). The substrate solution (BK) has a slight activity on *E. coli* but no activity on *S. aureus*.

Rutala et al. [57] showed that the use of soap and water can sometimes be less efficient due to their lower microbial reduction capacity (≤80% reduction, in comparison with a phenolic disinfectant, which offers 94–95% reduction) and also due to the possibility of contamination of the soap solution. However, a few hours later, the bacterial count was nearly back to the pretreatment level [57]. In an Ayliffe et al. study [57,58], bacterial contamination of soap and water without a disinfectant increased from 10 CFU/mL to 34,000 CFU/mL after cleaning a hospital ward. If the soap solution or the mop are reused, contamination will, in fact, be transferred from one room to another.

The use of strippable coatings offers the advantage of avoiding these re-contamination incidents like the ones described above because on each contaminated surface, a new coating is formed, and after removal, it can be compacted and sealed in small containers dedicated to biological waste. As a conventional substitute for the classical soap and water, disinfectants significantly improved microbial removal when a conventional string mop was used (95% vs. 68%) [57], but using a microfiber mop instead of the conventional mop could also prevent the possibility of transferring microbes from room to room if a new microfiber pad is used in each room. By comparing the classical decontamination methods with the advantages of the strippable coating method, coupled with the DF values obtained for our decontamination solutions, we can affirm that this new method, based on peelable films, ensures sufficiently high values of microbial reduction while bringing the advantage of consisting of eco-friendly materials.

To evaluate the efficacy of biological contaminant removal from the targeted surfaces, controlled contamination of Petri dishes with *E. coli* and *S. aureus* was performed, followed by the addition of the decontamination solution. At the end of the curing process, the obtained nanocomposite coatings were easily peed off (Figure 6), and the decontaminated surface was further investigated to evaluate the decontamination efficiency.

**Figure 9:**
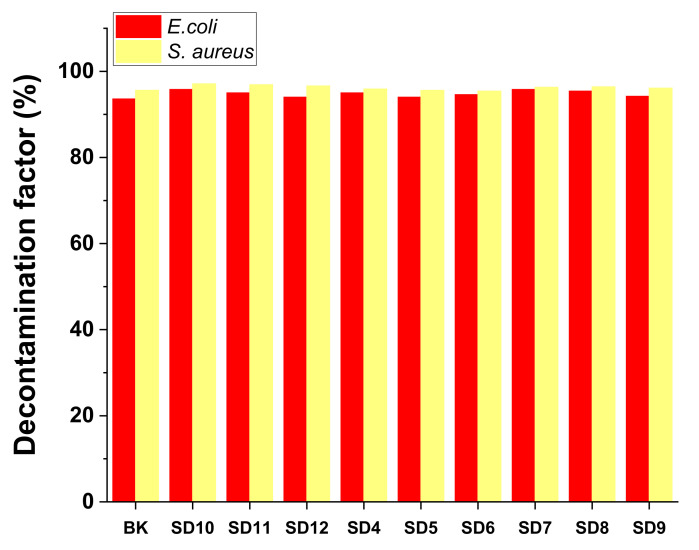
Biological decontamination efficacy.

The surfaces of Petri dishes were contaminated with portions of 10 µL suspension of *E. coli* (5 × 103 CFU/Petri dish) and *S. aureus* (7 × 103 CFU/Petri dish). Following the application of the decontamination solutions and removal of the peelable films, the number of residual micro-organisms on the targeted surface was assessed by cultivation in culture media (MHa). The effectiveness of the biological decontamination can be expressed utilizing the decontamination factor (DF). The decontamination factor can be calculated by the following equation:

DF = 100 × (C_i_−C_f_)/C_i_ (2)

where C_i_ represents the contamination level before applying the decontamination solution and C_f_ reflects the residual contamination [6]. Table 6 presents the DFs obtained for *E. coli* and *S. aureus*, and to facilitate comparison, Figure 9 summarizes all these values.

The efficacy of removal for the tested micro-organisms varies (93% < DF < 97%). Therefore, we can affirm that these polymeric decontamination solutions represent a useful tool for biological decontamination of surfaces. Basically, biological decontamination occurred through two mechanisms: the first one consists of the entrapment of micro-organisms in the polymeric matrix of the nanocomposite due to the excellent adsorptive properties of bentonite nanoclay; and the second one consists of the active inhibition of the activity of micro-organisms with the aid of the antimicrobial effect of the bentonite-supported Ag, Cu, and TiO_2_ nanoparticles present in the decontamination solutions. Therefore, even if some of the decontamination solutions did not show remarkable antimicrobial activity, they can still be successfully used for decontamination as they have a great potential for entrapping and sealing the biological contaminants inside the polymeric matrix of the nanocomposite film obtained following the evaporation of the solvent (water). The increased stability of the peelable films herein reported could ensure minimization of risks associated with biological contamination, ensuring immediate decontamination by first covering and then capturing the contaminant inside the polymeric film. The peeled nanocomposite films containing the entrapped contaminant can be further subjected to analysis for the identification and evaluation of the concentration of the contaminant.

### 3.3. Chemical Decontamination Tests

**Figure 10:**
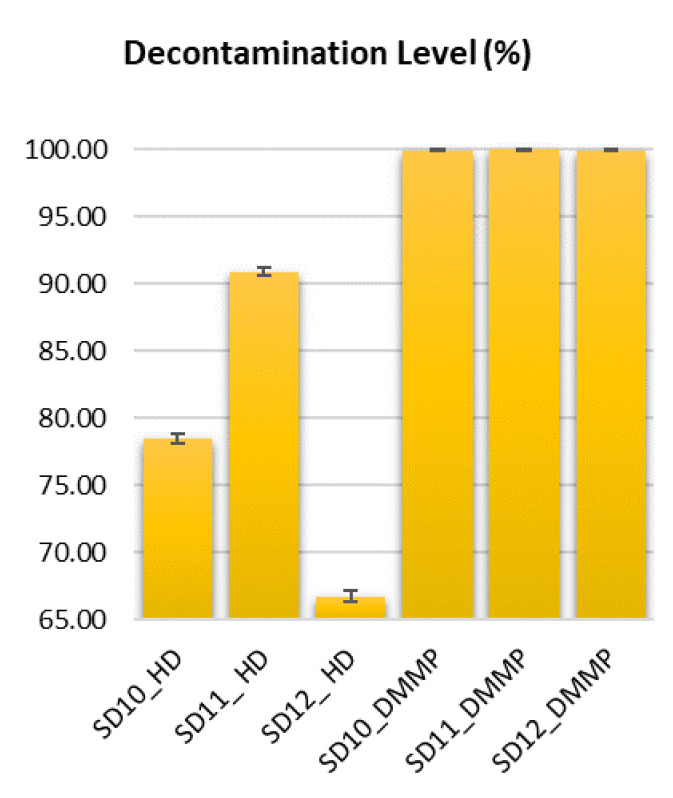
Chemical decontamination efficacy: decontamination factors.

Chemical decontamination tests followed the biological decontamination tests. Since the biological decontamination tests showed that the solutions based on bentonite-supported silver nanoparticles displayed the best results, we employed only these solutions (SD10, SD11, and SD12) for the tests performed on real chemical warfare agents. This choice was also influenced by safety concerns, as working with real warfare agents imposes higher risks and requires specially trained personal. Thus, we tried to limit the number of experiments by employing only these three decontamination solutions, and we maintained the relevant steps for the decontamination procedure in order to obtain accurate information. For the same reasons, we also tested a simulant. Chemical decontamination of metallic surfaces measuring 10 cm^2^ was accomplished in three stages: the first one consisted of the controlled contamination of the metallic coupons with **HD** and **DMMP** (10 mg/10 cm^2^), respectively; the second one consisted of applying the decontamination solution on the contaminated surface and allowing it to neutralize the toxin and to form the film by evaporation of the solvent at room temperature (25 °C); the last step consisted of DCM extraction of the decontaminated surface and of the peeled film. The results obtained for **HD** and **DMMP** are presented in Table 7, Figure 10. Some relevant chromatograms were selected and are shown in Figures S6–S9.

**Figure 11:**
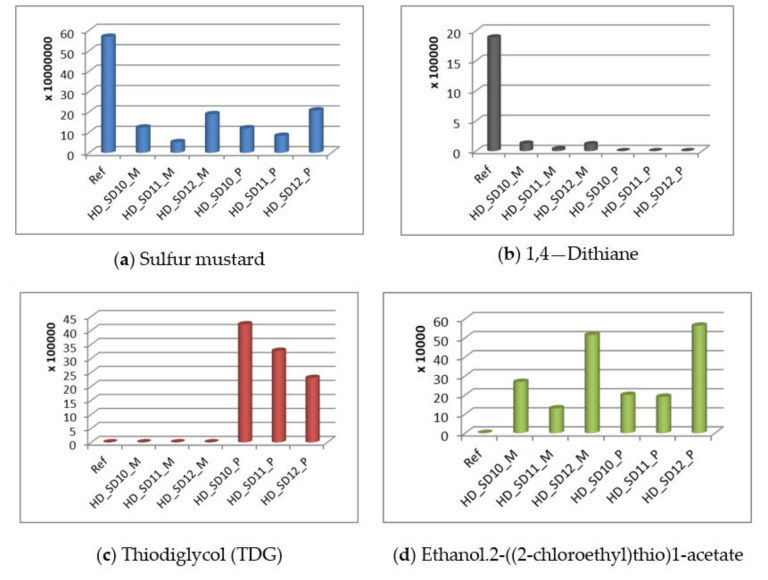

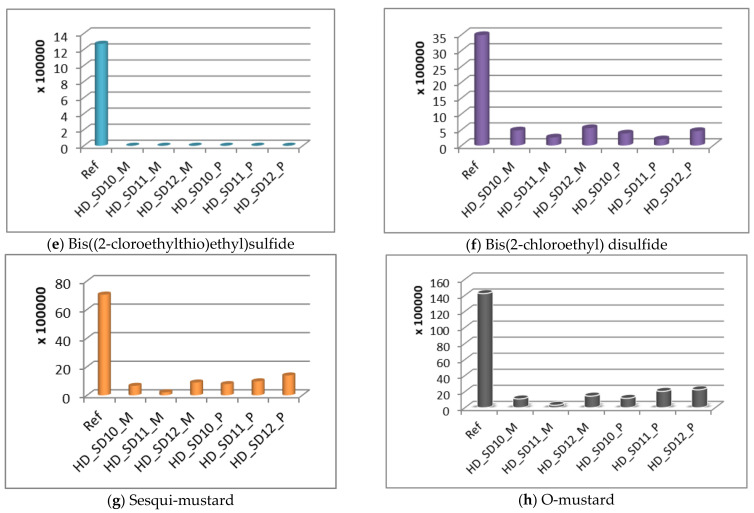
Degradation products of **HD** tracked during the decontamination process (*y*-axis values correspond to the values obtained for the area of the specific peak (counts) of each analyte): (**a**) sulfur mustard, (**b**) 1,4—dithiane, (**c**) thiodiglycol, (**d**) ethanol.2-((2-chloroethyl)thio)1-acetate, (**e**) Bis((2-cloroethylthio)ethyl)sulfide, (**f**) Bis(2-chloroethyl) disulfide, (**g**) sesqui-mustard and (**h**) O-mustard.

Decontamination factor was calculated according to the following equation, also described in the *Methods* section: DF = 100 × (C_0_ − C_f_)/C_0_, where DF is the decontamination factor, C_0_ is the initial toxic concentration found on the tested metallic surface, and C_f_ is the final concentration found on the decontaminated metallic surface, reflecting the residual contamination (according to the area of the characteristic peak of toxin). The values obtained are illustrated in Figure 11.

As it can be noticed in Table 7 and Figure 10, the SD11 decontamination solution achieved the highest decontamination factor for **HD**. In the case of **DMMP**, employed as simulant for nerve chemical agents, the decontamination factors obtained were much higher, with all decontamination solutions (SD10, SD11, and SD12) being highly efficient. It is well known that **HD** is more difficult to decontaminate due to its chemical structure, as it tends to establish stronger interactions with the metallic substrate on which it is deposited. Even so, SD11 managed to efficiently remove more than 90% from the contaminated surface. The other decontamination solutions were not so efficient, probably due to their composition. We can presume that SD10 was not able to entrap the same amount of toxin, probably due to the lower content of bentonite, which was reflected in a higher residual contamination ≈ 21.72%. On the other hand, even if SD12 had more bentonite and theoretically greater adsorptive capacity, having a slightly higher viscosity and less NP active centers (bigger NPs and lower specific surface) with decreased mobility in a more viscous media, this could have led to much lower DF values.

The achieved **HD** decontamination efficiency was 90.89% for SD11. This experiment demonstrates the functionality of the polymeric films used for the capture and removal of the toxic agent and also a small amount of waste generation. A comparative assessment between the result obtained and SD11 polymer films on **HD**-contaminated metallic surfaces (DF ≈ 90.89%) and conventional decontamination products, which are commercially available (bleach (full strength)- DF ≈ 86% or hydrogen peroxide- DF ≈ 71% for HD-contaminated metallic surfaces) [59], shows a clear improvement in terms of decontamination efficiency, afforded by the strippable coatings herein reported (SD11).

An ideal achievement is a 100% efficient decontamination, but in the case of operational decontamination, this percentage is relatively difficult to obtain for yperite, as the decontamination process depends on a multitude of factors, such as decontamination time, ambient temperature, the contact time elapsed between the contaminated surface and toxic agent until the application of the decontamination solution, and last but not least, the type of material that has been contaminated. In this regard, the U.S. Environmental Protection Agency (EPA) conducted some studies covering aspects of mustard decontamination using commercial decontamination solutions, wherein it concluded that depending on the factors listed above, decontamination efficiency can vary in practice between 37% and max. 95% when applying the decontamination product only once [59,60]. Thus, we can affirm that SD11 polymeric films present unique perspectives in their operational use for removing **HD** contaminant.

In general, **DMMP** is much easier to decontaminate because of the weaker interactions it establishes with the metallic surfaces on which it is deposited in decontamination tests. Even so, it still requires an adequate decontaminating agent. All the decontamination solutions managed to reach DF values greater than 99.96%. These high values of DF were obtained due to the remarkable adsorption capacity of the materials employed for decontamination and the compatibility of the components of the decontamination solutions with **DMMP** but also due to the weak interactions between **DMMP** and the metallic substrate.

The last step in the evaluation of chemical decontamination efficacy consisted of the investigation of the degradation products of sulfur mustard. The **HD** solution employed for controlled contamination had a purity of 95%. Thus, 5% of the solution contained by-products of the synthesis of **HD** and small amounts of degradation products (Table S5). The solution utilized for controlled contamination, together with the degradation products of **HD**, was also tracked during the decontamination process because part of these synthesis by-products from the **HD** initial solution is also part of the blister agent class, possessing a higher blistering action than neat **HD**. Thus, even if they are found in a small concentration in the initial solution, the higher toxicity of these compounds imposes the necessity of examining their degradation. The results are illustrated in Figure 11 and Table S5.

In Figure 11, it can be noticed that the decontamination solution does not just entrap the toxic, but it also actively decomposes **HD** (and the other initial components of the contaminating solution) into less toxic compounds. These results offer clear evidence of the ability of the decontamination solutions to efficiently neutralize the toxic agent. The decontamination is performed by two pathways: the chemical degradation of the toxins, which is possible with the aid of the active components, as well as the entrapment and sealing-off of the degradation products and the toxic compounds that were only partially degraded. As can be seen in Figure 11, **HD** was only partially degraded, as **HD** can still be detected on the surface and in the nanocomposite films after DCM extraction. sesqui-mustard and O–mustard, which are well-known for their higher toxicity, both present in the initial contaminating solution were also partially degraded. In comparison with **HD**, sesqui–mustard, and O-mustard, the compound 1,4—dithiane was not visible in the polymeric film after decontamination. Thiodiglycol, the hydrolysis product of **HD**, was found in significant quantities in the samples obtained from DCM extraction of the polymeric film after decontamination, thus offering evidence of the high capacity of these decontamination solutions to hydrolyze **HD**.

Based on the chemical decontamination tests, it can be concluded that these novel water-based decontamination solutions are a useful and versatile tool for the neutralization and removal of chemical warfare agents, ensuring high decontamination levels.

## 4. Conclusions

This study proposes new decontamination solutions consisting of innovative, ecological, peelable active nanocomposite films specially designed for biological and chemical warfare agents. These film-forming decontamination solutions are water-based solutions obtained from eco-friendly materials. Bentonite-supported nanoparticles (Cu, TiO_2_, and Ag) were successfully synthesized in aqueous solution and were employed in the decontamination formulations as active agents facilitating the neutralization of the hazardous materials. The unicity of these formulations consists of their environmentally responsible composition and high capacity to entrap and neutralize BCWA contaminants.

Particle-size control of the synthesized nanoparticles was accomplished by employing three different concentrations of bentonite nanoclay, which also served as adsorbent in the decontamination solutions, trapping the contaminants that diffuse in the polymeric composite network until the end of the drying process. Bentonite-supported silver nanoparticles displayed high antimicrobial activity and had a positive effect on the degradation process of the chemical warfare agent sulfur mustard, as well as **DMMP**, a nerve agent simulant. TEM analyses confirmed the nanometric dimensions of the obtained metallic particles. The decontamination formulations were further prepared based on these active ingredients and a water-soluble polymer, APV. Their viscosity was evaluated, revealing only minor differences between them due to the low concentration of nanoparticles and nanoclay (up to 1.5%). Viscosity influences the application method (spraying vs. brushing), but it also influences the motion of the active ingredients towards the contaminants within the polymeric matrix. The evaporation rate of each decontamination solution was evaluated to assess the necessary time for obtaining the peelable nanocomposite films. Chemical, mechanical, and thermal characterizations of the polymeric nanocomposite films were performed using FT-IR, tensile tests, DTA, and DMA techniques, showing the influence of each component on the final properties of the polymeric nanocomposite designed for BCWA decontamination. The decontamination effectiveness was first evaluated by qualitative and quantitative approaches, employing specific analytic tools for each type of contaminant. The influence of the concentration of bentonite nanoclay, and subsequently, the influence of the nature and size of the synthesized nanoparticles over the decontamination efficiency were also emphasized. The presence of nanoparticles led to higher decontamination factors. The solutions containing Ag-NPs displayed more antimicrobial activity. Copper nanoparticles displayed less antimicrobial activity, but this aspect was improved by the addition of TiO_2_ nanoparticles. The efficacy of removal for the tested micro-organisms varies (93% < DF < 97%), thus confirming that these polymeric decontamination solutions represent a useful tool for biological decontamination of surfaces. The decontamination solution containing 1% bentonite nanoclay and Ag-NPs (**SD11**) displayed the best results for **HD** decontamination (DF ≥ 90.89%). In contrast, **DMMP** was almost completely removed from the contaminated surfaces, displaying a decontamination factor of DF ≈ 99.97% ± 0.01.

In conclusion, the eco-friendly, peelable active nanocomposite films designed for biological and chemical warfare agent decontamination can be successfully used on contaminated surfaces, reducing the risk of spreading bio-contaminants or chemical agents by neutralizing and entrapping the hazardous materials and their degradation products into the polymer nanocomposite matrix. In comparison with classical decontamination methods, employing ecological peelable coatings brings multiple advantages: significantly lower consumption of water and reagents, significantly lower amount of post-decontamination waste, ease of application, eco-friendly components, and high decontamination factors for both biological and chemical agents.

## Figures and Tables

**Table 1:** Composition of the reference samples.

Sample	PVA (wt.%)	GLY (wt.%)	BT (wt.%)
SD1	10	-	-
SD2	10	5	-
SD3	10	5	1

**Table 2:** Composition of the solutions containing CuNPs.

Sample	PVA (wt.%)	GLY (wt.%)	BT (wt.%)	Cu (CH_3_COO)_2_ • H_2_O (wt.%)	TiO_2_ (wt.%)	*Dispersing Agent* Triton X100 (wt.%)	*Reducing Agent* Ascorbic Acid (wt.%)
SD4	10	5	0.5	0.04	-	-	0.143
SD5	10	5	1	0.04	-	-	0.143
SD6	10	5	1.5	0.04	-	-	0.143
SD7	10	5	0.5	0.04	1	0.25	0.143
SD8	10	5	1	0.04	1	0.25	0.143
SD9	10	5	1.5	0.04	1	0.25	0.143

**Table 3:** Composition of the solutions containing AgNPs.

Sample	PVA (wt.%)	GLY (wt.%)	BT (wt.%)	AgNO_3_ (wt.%)	*Reducing Agent 1* Trisodium Citrate Dihydrate (wt.%)	*Reducing Agent 2* NaBH_4_ (wt.%)
SD10	10	5	0.5	0.04	0.012	0.01
SD11	10	5	1	0.04	0.012	0.01
SD12	10	5	1.5	0.04	0.012	0.01

**Table 4:** Minimal inhibitory concentration (MIC) and Minimal bactericidal concentration (MBC).

Sample/ Micro-Organism	*E. coli*		*Ps. aeruginosa*		*S. aureus*	
	MIC (%)	MBC (%)	MIC (%)	MBC (%)	MIC (%)	MBC (%)
SD4	>50	--	50	--	50	--
SD5	>50 *	--	50	--	50	--
SD6	>50 *	--	50	--	25	--
SD7	>50 *	--	25	--	1.56	25
SD8	50	--	25	--	0.78	12.5
SD9	25	--	25	--	0.39	25
SD10	6.25	25	0.39	3.125	3.125	--
SD11	12.5	25	0.78	12.5	6.25	--
SD12	12.5	25	0.39	6.25	6.25	--
SD3 (BK)	--	--	--	--	--	--

* Under the test conditions, the antimicrobial activity of the solution against the *E. coli* micro-organism could not be highlighted.

**Table 5:** Bacterial cell population decrease (%) after 2 h of contact with decontamination solutions.

Micro-Organism/Sample	SD4	SD5	SD6	SD7	SD8	SD9	SD10	SD11	SD12	SD3 (BK)
*E. coli*	96.25	98.65	97.53	98.1	97.23	93.25	95.43	95.43	80.25	47.5
*S. aureus*	87.2	93.7	86.6	90.4	94.6	95.8	96.1	97	97.35	−75

**Table 6:** Efficacy of removal of *E. coli* and *S. aureus* strains from surfaces.

Micro-Organism/Sample	SD4	SD5	SD6	SD7	SD8	SD9	SD10	SD11	SD12	SD3 (BK)
*E. coli*	95	94	94.6	95.8	95.4	94.2	95.8	95	94	93.6
*S. aureus*	95.9	95.6	95.4	96.3	96.4	96.1	97.1	96.9	96.6	95.6

**Table 7:** Evaluation of chemical decontamination efficacy with the aid of GC-MS results.

Sample ID	DF (%)	Residual Contamination (%)	Residual Contamination (mg/10 cm^2^)
S0_HD	0	100	10
HD_SD10_M	78.28	21.72	2.1722
HD_SD11_M	90.89	9.11	0.9111
HD_SD12_ M	66.73	33.27	3.3273
HD_SD10_P	N/A	21.02	2.1018
HD_SD11_P	N/A	14.58	1.4579
HD_SD12_P	N/A	36.47	3.6470
S0_DMMP	0	100	10
DMMP_SD10_M	99.97	0.03	0.0026
DMMP_SD11_M	99.98	0.02	0.0018
DMMP_SD12_ M	99.96	0.04	0.0036
DMMP_SD10_P	N/A	3.00	0.2619
DMMP_SD11_P	N/A	5.79	0.5063
DMMP_SD12_P	N/A	7.53	0.6584

**HD**—sulfur mustard; S0_HD—sulfur mustard blank; **DMMP**—dimethyl methylphosphonate; S0_**DMMP**—dimethyl methylphosphonate blank; M—samples extracted in DCM from the metalic surfaces after decontamination; P—samples extracted in DCM from the nanocomposite film after decontamination.

## Data Availability

The data presented in this study are available on request from the corresponding author.

## Supplementary Materials

The following are available online at https://www.mdpi.com/article/10.3390/polym13223999/s1, Table S1. Evaporation rate of the decontamination solutions; Figure S1. FT-IR spectra of the polymer nanocomposite films; Figure S2. Tensile tests results; Table S2. Tensile tests results; Figure S3. Cell population after 2 h in contact with the decontamination solutions for *E. coli*; Table S3. Antimicrobial activity of the decontamination solutions against *E. coli*; Figure S4. Cell population after 24 h in contact with the decontamination solutions for *E. coli*; Table S4. Antimicrobial activity of the decontamination solutions against *S. aureus*; Figure S5. Mass spectra of **HD** (up—from analysis; down—NIST database) RT—10.71 min; Figure S6. Chromatograms multigraph; Figure S7. Total Ions Chromatograms overlap for all samples, representing **HD** at the RT (retention time): 10.70; Figure S8. Chromatograms overlap for reference sample and samples extracted from the metallic surface after decontamination; Figure S9. Chromatograms overlap for reference sample and samples extracted from the nanocomposite film after decontamination; Table S5. Degradation products of **HD** monitorized during the decontamination process; Figure S10. Degradation products of Yperite; Figure S11. Mass spectra of **HD** (up—from analysis; down—NIST database) RT—5.93 min; Figure S12. Chromatograms overlap for all samples; Figure S13. Chromatograms overlap for all samples; Figure S14. Chromatograms overlap for reference sample and samples extracted from the metallic surface after decontamination; Figure S15. Chromatograms overlap for reference sample and samples extracted from the nanocomposite film after decontamination.
