# Supplementary material for: Eco–Friendly Peelable Active Nanocomposite Films Designed for Biological and Chemical Warfare Agents Decontamination
Source: Polymers (Basel). 2021 Nov 19;13(22):3999. doi: 10.3390/polym13223999 (PMC8620509; doi:10.3390/polym13223999)
Supplement: Supplementary file 1 [file polymers-13-03999-s001.zip › polymers-1457884-supplementary.pdf]

# Supporting Info

## Eco-friendly peelable active nanocomposite films designed for Biological and Chemical Warfare Agents decontamination

Gabriela Toader [1, ‡], Aurel Diacon [2, ‡], Traian Rotariu [1,*], Mioara Alexandru [3,*], Edina Rusen [2], Raluca Elena Ginghină [4], Florentina Alexe [4], Ramona Oncioiu [4], Florina Lucica Zorila [3], Alice Podaru [1], Andreea Elena Moldovan [1], Daniela Pulpea [1], Ana Mihaela Gavrilă [5], Tanta Verona Iordache [5] and Raluca Șomoghi [5, 6]

[1] Military Technical Academy "Ferdinand I", 39-49 George Cosbuc Boulevard, 050141 Bucharest, Romania;
[2] Faculty of Applied Chemistry and Materials Science, University 'Politehnica' of Bucharest, 1-7 Gh. Polizu Street, 011061 Bucharest, Romania;
[3] Microbiology Laboratory of Horia Hulubei National Institute for R&D in Physics and Nuclear Engineering, 30 Reactorului St., 077125 Bucharest – Măgurele, Romania;
[4] Research and Innovation Center for CBRN Defense and Ecology, 225 Soseaua Oltenitei, 041327 Bucharest, Romania;
[5] National Institute of Research and Development for Chemistry and Petrochemistry, 202 Splaiul Independentei, 060041 Bucharest, Romania;
[6] Petroleum-Gas University of Ploiesti, 100680 Ploiesti, Romania

*Correspondence: traian.rotariu@mta.ro (T.R.), mioara.alexandru@nipne.ro (M.A.)
‡ Co-first authors, equally contributed to this study

**Table S1.** *Evaporation rate of the decontamination solutions*

| Sample | Evaporation rate (%wt./h) | | | |
|---|---|---|---|---|
| | **25 $^{O}$C** | **30 $^{O}$C** | **35 $^{O}$C** | **40 $^{O}$C** |
| **SD1** | 5.5 | 10.2 | 15.4 | 23.0 |
| **SD2** | 5.5 | 10.1 | 15.6 | 21.4 |
| **SD3** | 5.5 | 9.6 | 13.1 | 22.5 |
| **SD4** | 4.9 | 8.6 | 14.4 | 20.6 |
| **SD5** | 5.5 | 8.5 | 14.7 | 21.5 |
| **SD6** | 5.4 | 9.8 | 15.2 | 21.8 |
| **SD7** | 5.3 | 10.4 | 15.9 | 23.3 |
| **SD8** | 6.0 | 11.4 | 16.9 | 23.3 |
| **SD9** | 6.1 | 10.4 | 15.6 | 21.9 |
| **SD10** | 6.2 | 10.6 | 16.5 | 22.2 |
| **SD11** | 5.0 | 10.0 | 16.0 | 23.7 |
| **SD12** | 5.6 | 10.2 | 16.1 | 23.6 |

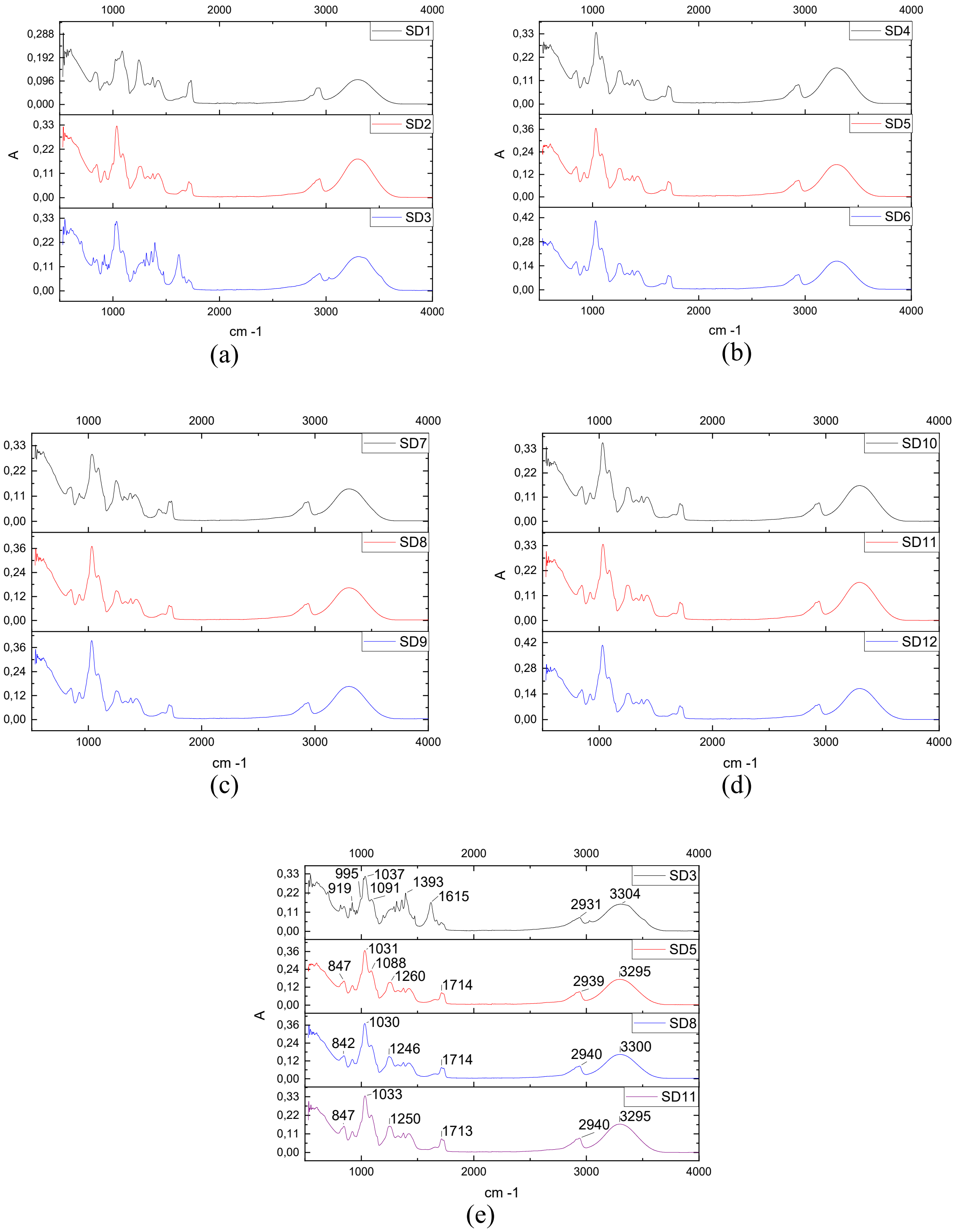


**Figure S1.** FT-IR spectra of the polymer nanocomposite films.

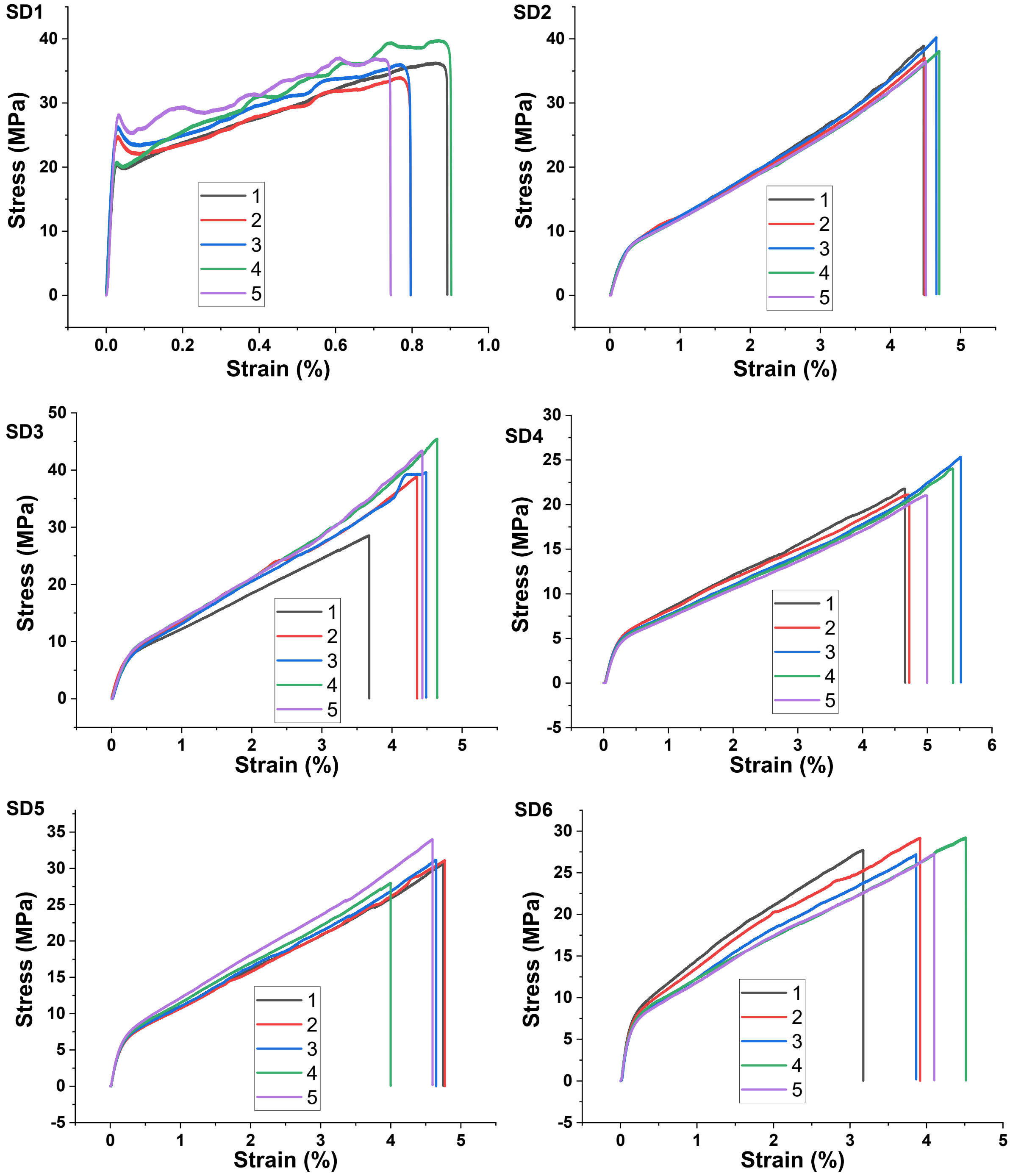
SD1
Stress (MPa)
Strain (%)
1
2
3
4
5
SD2
Stress (MPa)
Strain (%)
1
2
3
4
5
SD3
Stress (MPa)
Strain (%)
1
2
3
4
5
SD4
Stress (MPa)
Strain (%)
1
2
3
4
5
SD5
Stress (MPa)
Strain (%)
1
2
3
4
5
SD6
Stress (MPa)
Strain (%)
1
2
3
4
5

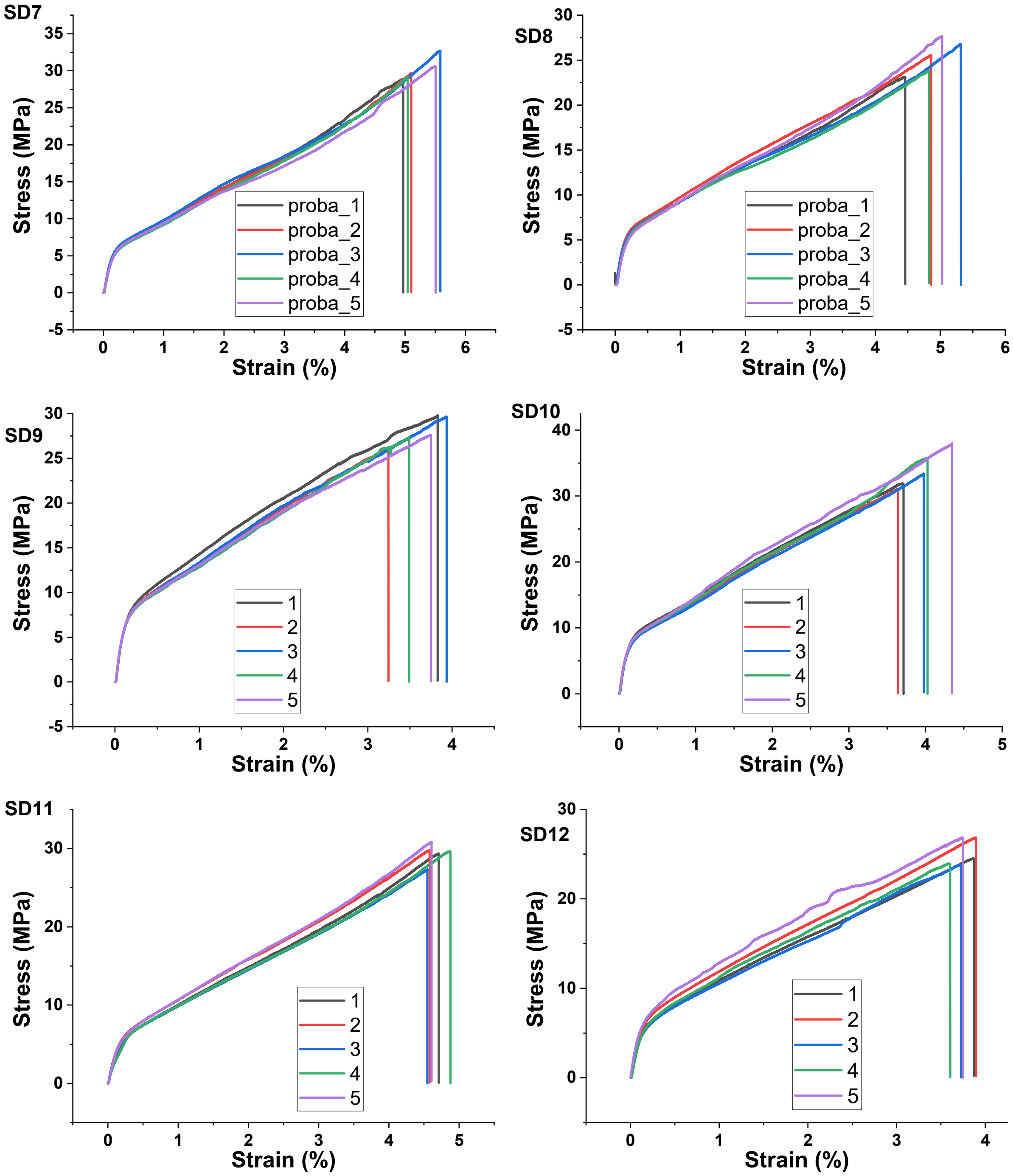


**Figure S2.** Tensile tests results.

**Table S2.** Tensile tests results.

| Sample | $\varepsilon_{max}$ (%) | $\sigma_{max}$ (MPa) | Young modulus (MPa) |
|---|---|---|---|
| **SD1** | 0.8769 | 36.0593 | 41.123 |
| **SD2** | 4.6537 | 40.1990 | 8.638 |
| **SD3** | 4.4317 | 43.3565 | 9.783 |
| **SD4** | 4.9943 | 20.9684 | 4.198 |
| **SD5** | 4.5949 | 33.9905 | 7.397 |
| **SD6** | 4.0999 | 27.2418 | 6.644 |
| **SD7** | 5.0431 | 29.1408 | 5.778 |
| **SD8** | 4.8274 | 23.8357 | 4.938 |
| **SD9** | 3.7492 | 27.6316 | 7.370 |
| **SD10** | 4.6081 | 30.8314 | 6.691 |
| **SD11** | 3.9761 | 33.3954 | 8.399 |
| **SD12** | 3.8693 | 24.5156 | 6.336 |

**Biological decontamination tests**

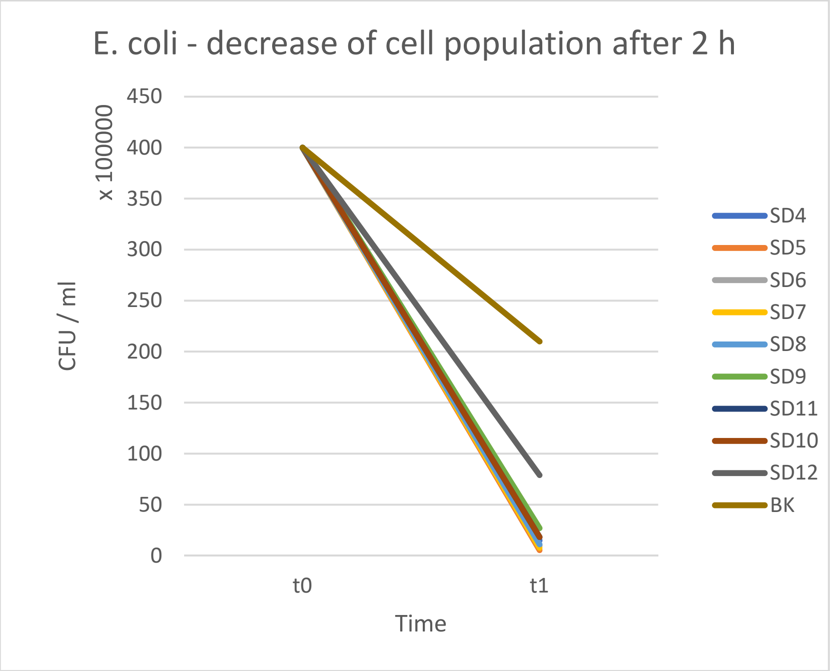


**Figure S3.** Cell population after 2 hours in contact with the decontamination solutions for *E coli.*

**Table S3.** Antimicrobial activity of the decontamination solutions against *E.coli.*

| | SD4 (UFC / mL) | SD5 (UFC / mL) | SD6 (UFC / mL) | SD7 (UFC / mL) | SD8 (UFC / mL) | SD9 (UFC / mL) | SD10 (UFC / mL) | SD11 (UFC / mL) | SD12 (UFC / mL) | SD3 (UFC / mL) |
|---|---|---|---|---|---|---|---|---|---|---|
| **$T_0$** ($10^5$ UFC / ml) | 52 × $10^5$ | 65 × $10^5$ | 50 × $10^5$ | 45 × $10^5$ | 65 × $10^5$ | 200 × $10^5$ | 59 × $10^5$ | 53 × $10^5$ | 49 × $10^5$ | 280 × $10^5$ |
| **$T_1$** ($10^5$ UFC / mL) | 4.5 × $10^5$ | 5.1 × $10^5$ | 3.8 × $10^5$ | 8.9 × $10^5$ | 50 × $10^5$ | 100 × $10^5$ | 43 × $10^3$ | 35 × $10^3$ | 25 × $10^3$ | 250 × $10^5$ |

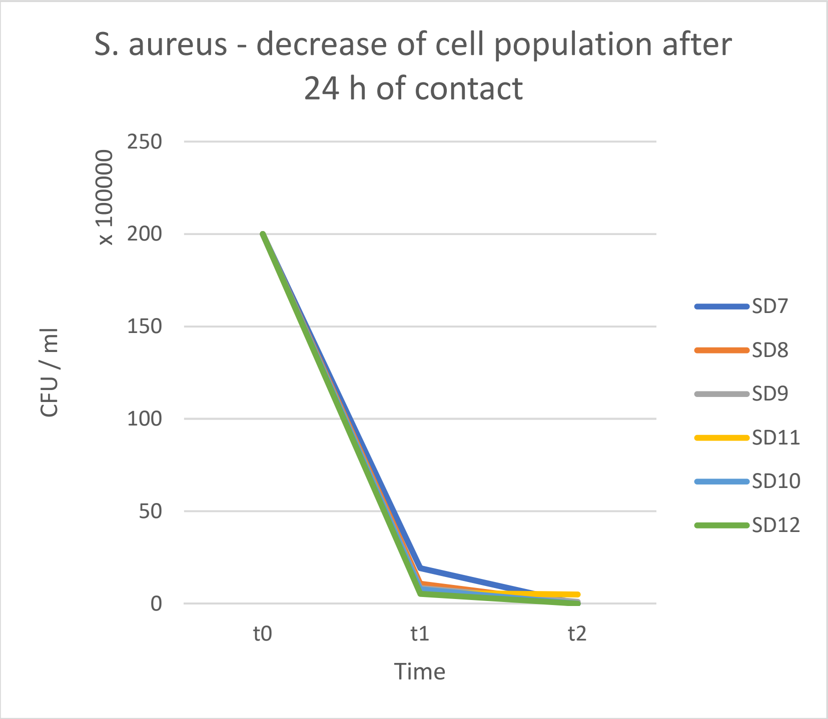


**Figure S4.** Cell population after 24 hours in contact with the decontamination solutions for *E coli.*

**Table S4.** Antimicrobial activity of the decontamination solutions against *S. aureus.*

| | SD4 (UFC / mL) | SD5 (UFC / mL) | SD6 (UFC / mL) | SD7 (UFC / mL) | SD8 (UFC / mL) | SD9 (UFC / mL) | SD10 (UFC / mL) | SD11 (UFC / mL) | SD12 (UFC / mL) | SD3 (UFC / mL) |
|---|---|---|---|---|---|---|---|---|---|---|
| **$T_0$** ($10^5$ UFC / mL) | 320 × $10^5$ | 290 × $10^5$ | 90 × $10^5$ | 40 × $10^5$ | 32 × $10^5$ | 290 × $10^5$ | 250 × $10^5$ | 270 × $10^5$ | 290 × $10^5$ | 300 × $10^5$ |
| **$T_1$** ($10^5$ UFC / mL) | 289 × $10^5$ | 66 × $10^5$ | 43 × $10^5$ | < $10^2$ | < $10^2$ | $10^3$ | 2 × $10^3$ | < $10^2$ | < $10^2$ | 290 × $10^5$ |

## Chemical Decontamination tests

### Sulphur mustard

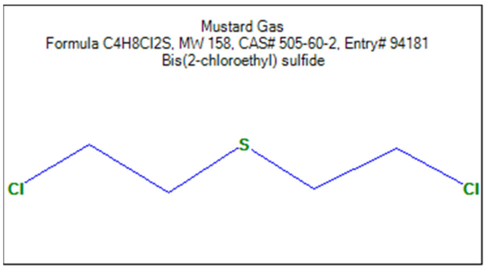


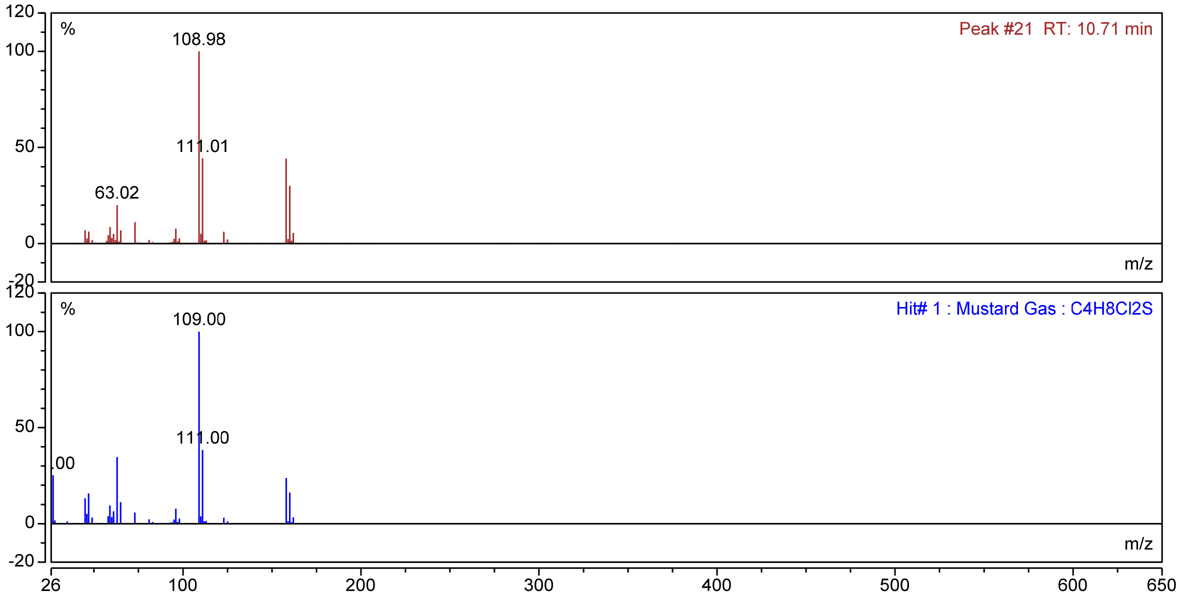


**Figure S5.** Mass spectra of HD (up – from analysis; down – NIST database), RT – 10.71 min.

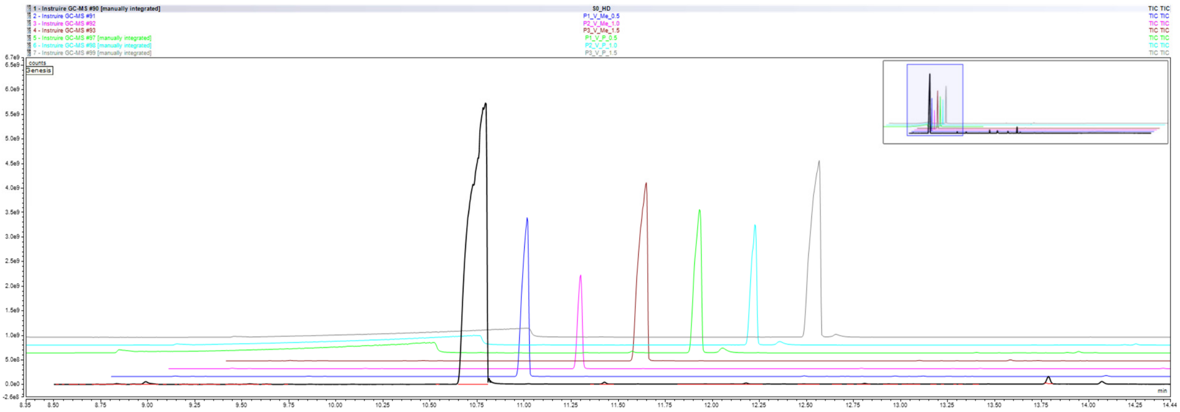

**Figure S6.** Chromatograms multigraph.

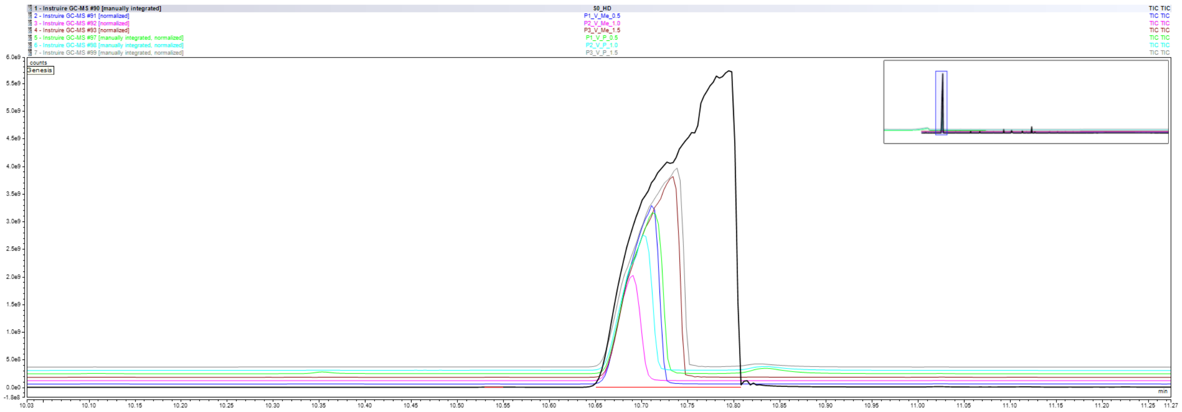

**Figure S7.** Total Ions Chromatograms overlap for all samples, representing HD at the RT (retention time): 10.70;

(blank - initial HD contamination; blue - residual HD after decontamination with SD10, extracted with DCM from the metallic surface; magenta - residual HD after decontamination with SD11, extracted with DCM from the metallic surface; burgundy -residual HD after decontamination with SD12, extracted with DCM from the metallic surface; green – unreacted HD found after decontamination in peeled film SD10, extracted with DCM; cyan - unreacted HD found after decontamination in peeled film SD11, extracted with DCM; gray - unreacted HD found after decontamination in peeled film SD12, extracted with DCM).

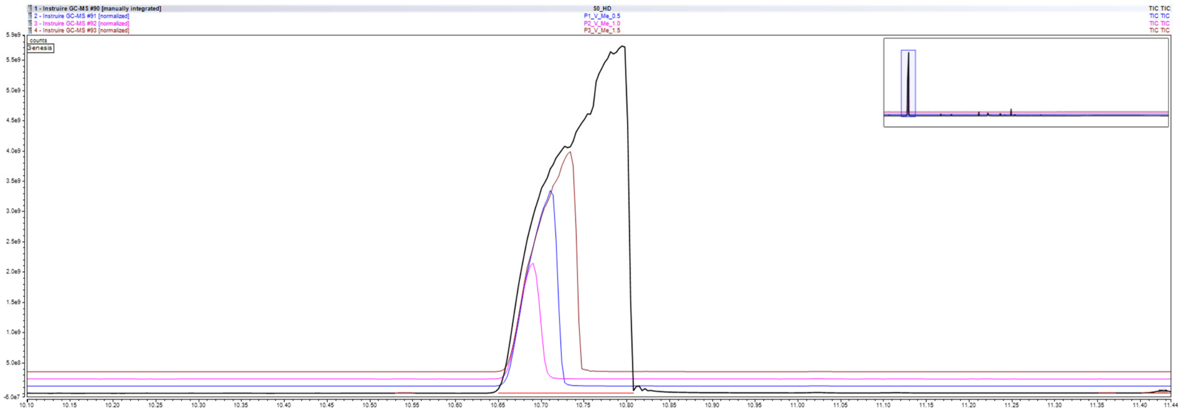

**Figure S8.** Chromatograms overlap for reference sample and samples extracted from the metallic surface after decontamination.

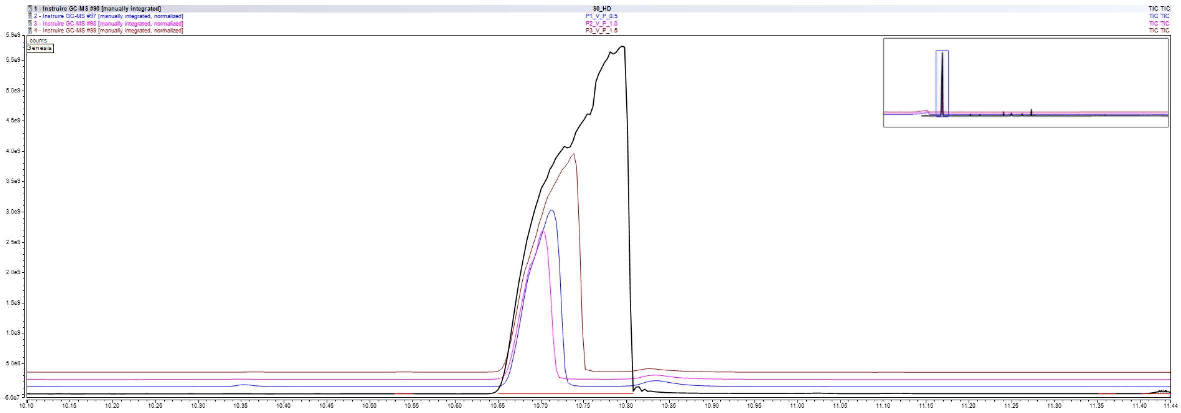

**Figure S9.** Chromatograms overlap for reference sample and samples extracted from the nanocomposite film after decontamination.

**Table S5.** Degradation products of HD monitorized during the decontamination process.

| No | Compound | RT | AS0 | AP1Me | AP2Me | AP3Me | AP1P | AP2P | AP3P |
|---|---|---|---|---|---|---|---|---|---|
| 1 | Dithiane | 8.99 | 1887741 | 124343 | 32416 | 114606 | 0 | 0 | 0 |
| 2 | HD | 10.79 | 568555828 | 12350046 1 | 51799820 | 189174524 | 119498537 | 82888058 | 207352784 |
| 3 | Thiodiglycol (TDG) | 10.84 | 0 | 0 | 0 | 0 | 4218280 | 3269076 | 2301103 |
| 4 | Etanol 2-((2-cloroetil)tio) 1-acetate | 12.67 | 0 | 268287 | 129480 | 515104 | 200100 | 190421 | 563526 |
| 5 | Bis(2-cloroetil) disulfide | 13.78 | 3485028 | 475053 | 252378 | 547932 | 378536 | 194092 | 453803 |
| 6 | Bis((2-cloroetiltio)etil)sulfide | 14.07 | 1270521 | 0 | 0 | 0 | 0 | 0 | 0 |
| 7 | Sesqui-mustard | 17.33 | 7045057 | 662471 | 208319 | 895429 | 782569 | 978155 | 1386415 |
| 8 | O-mustard | 20.33 | 14223291 | 1097079 | 314214 | 1449068 | 1172060 | 2040661 | 2238374 |

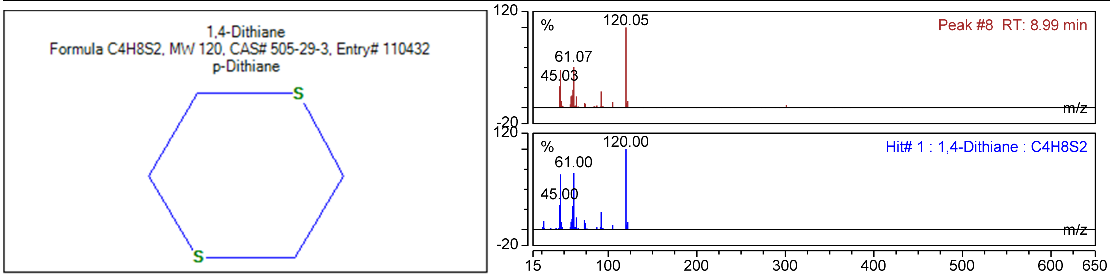


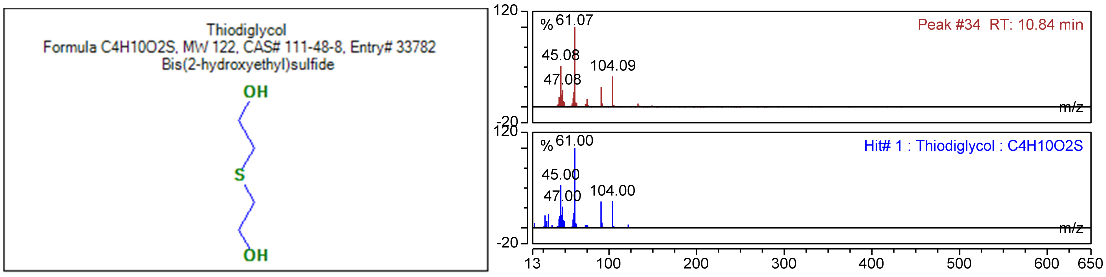

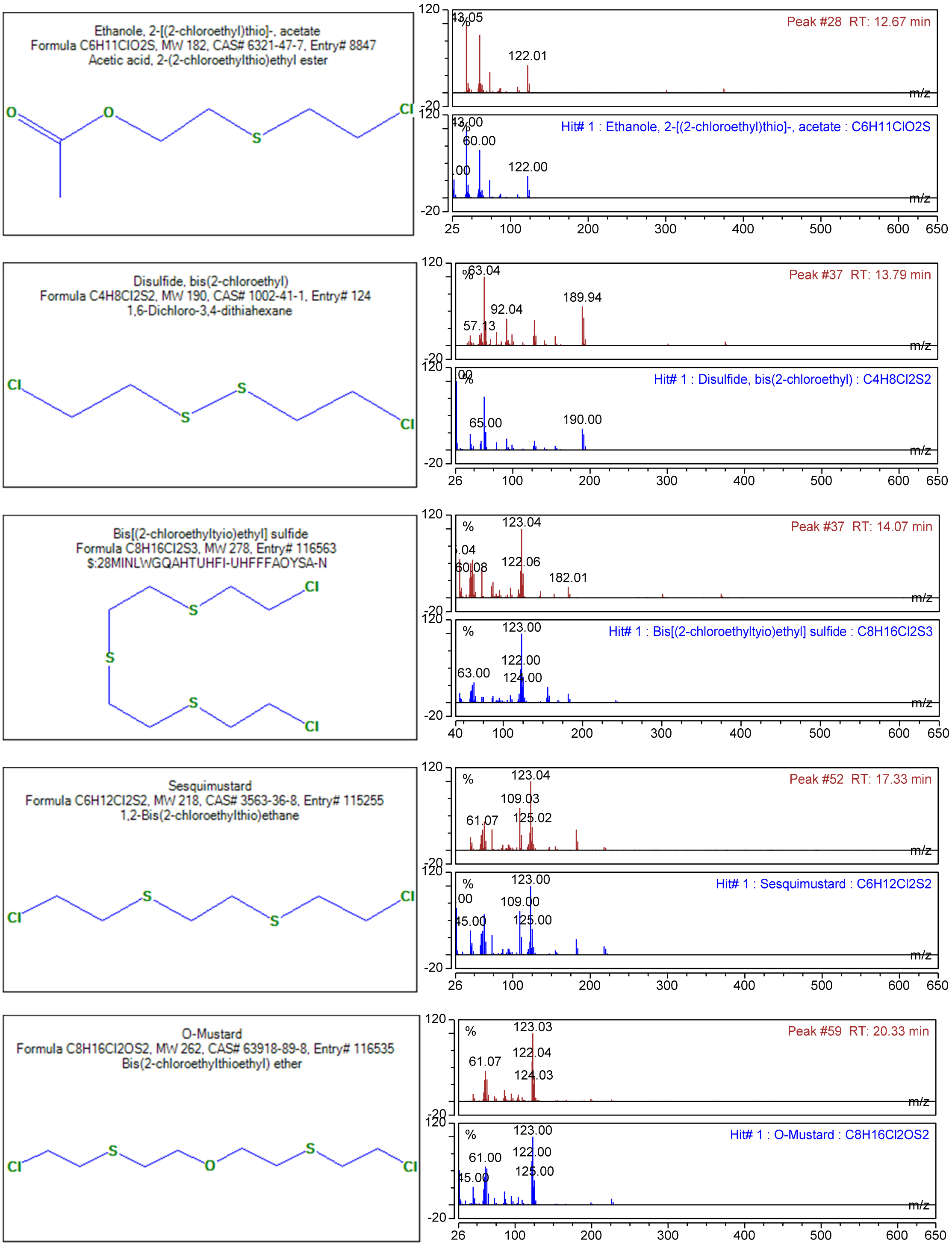


**Figure S10.** Degradation products of Yperite.

## Dimethyl methylphosphonate

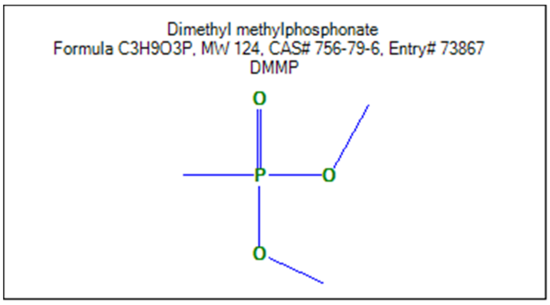


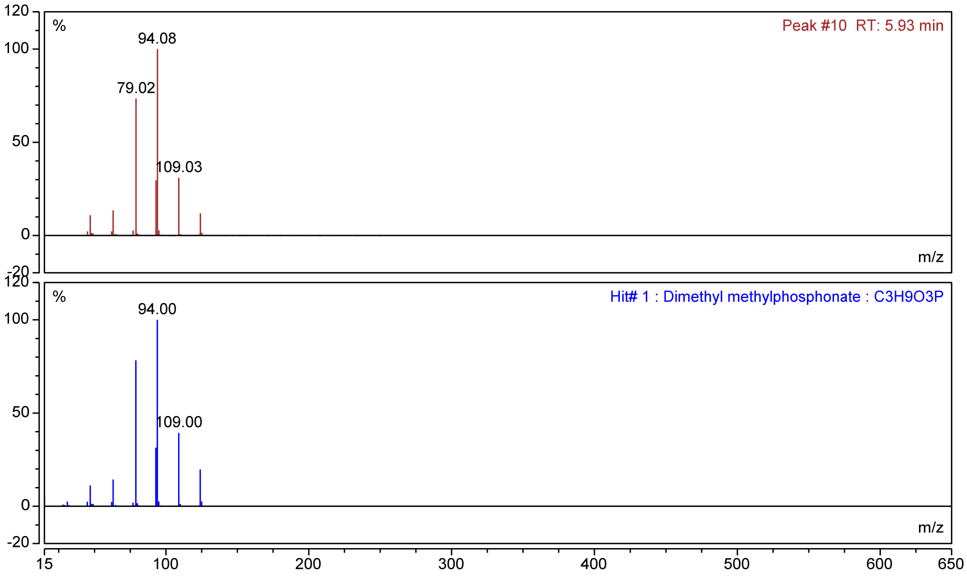


**Figure S11.** Mass spectra of HD (up – from analysis; down – NIST database) RT – 5.93 min.

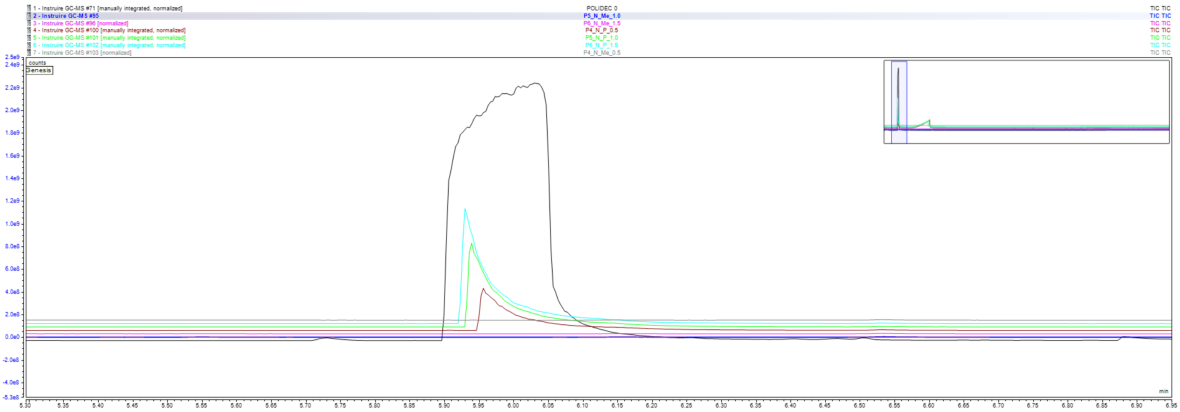

**Figure S12.** Chromatograms overlap for all samples.

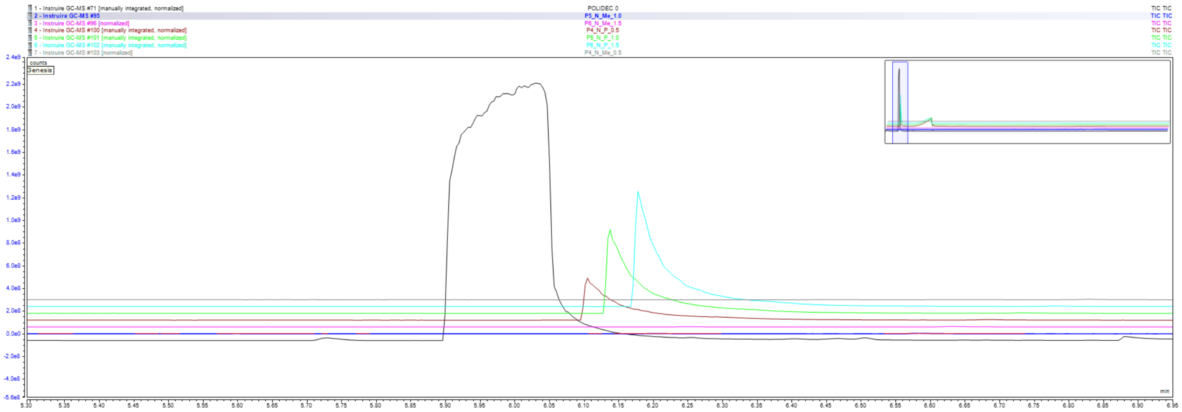

**Figure S13.** Chromatograms overlap for all samples.

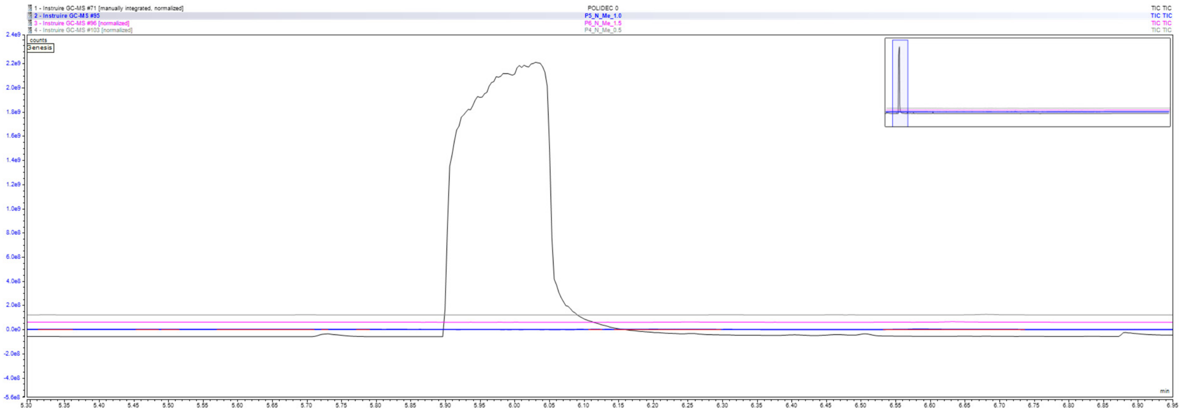

**Figure S14.** Chromatograms overlap for reference sample and samples extracted from the metallic surface after decontamination.

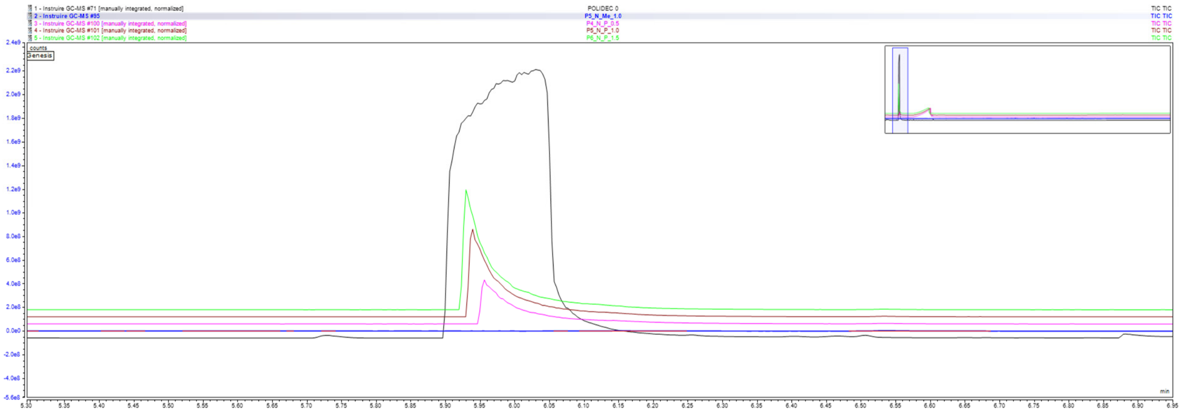

**Figure S15.** Chromatograms overlap for reference sample and samples extracted from the nanocomposite film after decontamination.
